# PKP2 orchestrates OXPHOS expression in cardiomyocytes via a PGC1α-dependent mechanism

**DOI:** 10.1093/cvr/cvag141

**Published:** 2026-10-10

**Authors:** Su Ji Han, Daphne van den Berg, Hesther de Ruiter, Hoyee Tsui, Eirini Kyriakopoulou, Tim Koopmans, Ilaria Perini, Jantine Monshouwer-Kloots, Sebastiaan J van Kampen, Anneline S J M te Riele, Ilse R Kelters, Monica Gianoli, Boudewijn M T Burgering, Eva van Rooij

**Affiliations:** Hubrecht Institute, Royal Netherlands Academy of Arts and Sciences (KNAW) and University Medical Center Utrecht, Uppsalalaan 8, Utrecht 3584 CT, the Netherlands; Hubrecht Institute, Royal Netherlands Academy of Arts and Sciences (KNAW) and University Medical Center Utrecht, Uppsalalaan 8, Utrecht 3584 CT, the Netherlands; Hubrecht Institute, Royal Netherlands Academy of Arts and Sciences (KNAW) and University Medical Center Utrecht, Uppsalalaan 8, Utrecht 3584 CT, the Netherlands; Hubrecht Institute, Royal Netherlands Academy of Arts and Sciences (KNAW) and University Medical Center Utrecht, Uppsalalaan 8, Utrecht 3584 CT, the Netherlands; Hubrecht Institute, Royal Netherlands Academy of Arts and Sciences (KNAW) and University Medical Center Utrecht, Uppsalalaan 8, Utrecht 3584 CT, the Netherlands; Hubrecht Institute, Royal Netherlands Academy of Arts and Sciences (KNAW) and University Medical Center Utrecht, Uppsalalaan 8, Utrecht 3584 CT, the Netherlands; Hubrecht Institute, Royal Netherlands Academy of Arts and Sciences (KNAW) and University Medical Center Utrecht, Uppsalalaan 8, Utrecht 3584 CT, the Netherlands; Hubrecht Institute, Royal Netherlands Academy of Arts and Sciences (KNAW) and University Medical Center Utrecht, Uppsalalaan 8, Utrecht 3584 CT, the Netherlands; Hubrecht Institute, Royal Netherlands Academy of Arts and Sciences (KNAW) and University Medical Center Utrecht, Uppsalalaan 8, Utrecht 3584 CT, the Netherlands; Department of Cardiology, Division of Heart and Lungs, University Medical Center Utrecht, Utrecht 3584 CX, the Netherlands; Experimental Cardiology Laboratory, Division of Heart and Lungs, University Medical Center Utrecht, Utrecht 3584 CX, the Netherlands; Department of Cardiothoracic Surgery, Division of Heart and Lungs, University Medical Center Utrecht, Utrecht 3584 CX, the Netherlands; Molecular Cancer Research, University Medical Center Utrecht, Utrecht 3584 CX, the Netherlands; Hubrecht Institute, Royal Netherlands Academy of Arts and Sciences (KNAW) and University Medical Center Utrecht, Uppsalalaan 8, Utrecht 3584 CT, the Netherlands; Department of Cardiology, Division of Heart and Lungs, University Medical Center Utrecht, Utrecht 3584 CX, the Netherlands

**Keywords:** Plakophilin-2 (PKP2), Arrhythmogenic cardiomyopathy (ACM), Oxidative phosphorylation, PGC1α, Cardiomyocyte, Mitochondrial function

## Abstract

**Aims:**

Arrhythmogenic cardiomyopathy (ACM) is an inherited cardiac disease in which most patients carry (likely) pathogenic variants in desmosomal genes, most commonly in plakophilin-2 (*PKP2*). While the genetic causes are well described, the molecular mechanisms driving disease progression—particularly in response to exercise—remain poorly understood.

**Methods and results:**

To investigate disease-driving pathways in ACM, we used *PKP2* mutant human-induced pluripotent stem cell-derived cardiomyocytes (hiPSC-CMs) and myocardial tissue from ACM patients. Transcriptomic profiling revealed consistent downregulation of oxidative phosphorylation (OXPHOS) genes in *PKP2* mutant hiPSC-CMs, which was confirmed in explanted ACM hearts carrying *PKP2* variants. Reduced expression of OXPHOS-related genes was associated with lower *PPARGC1A* expression and decreased mitochondrial spare capacity in *PKP2* mutant hiPSC-CMs. The reduction in *PPARGC1A* expression appeared to result, at least in part, from decreased AMPKα protein levels, a known activator of *PPARGC1A* previously shown to localize at the intercalated disc, as *PKP2* mutant cells failed to increase *PPARGC1A* expression following treatment with AICAR, an AMPK activator. To directly assess whether increasing *PPARGC1A* expression could compensate for this defect, we overexpressed *PPARGC1A*, which partially restored OXPHOS gene expression and improved contractility in *PKP2* mutant cells.

**Conclusion:**

Our study identifies a novel transcriptional mechanism linking PKP2 loss to reduced mitochondrial function via decreased PGC1α signaling, partially through reduced AMPKα levels. Disruption of this axis reduces OXPHOS gene expression and mitochondrial function in *PKP2* mutant hiPSC-CMs, potentially impairing the heart’s ability to meet increased energy demands during stress, and offering a mechanistic explanation for the detrimental effects of exercise in ACM. Furthermore, these findings highlight PGC1α induction as a potential therapeutic strategy to restore OXPHOS gene expression and mitochondrial adaptability in ACM.

## Graphical Abstract

**Graphical Abstract:**
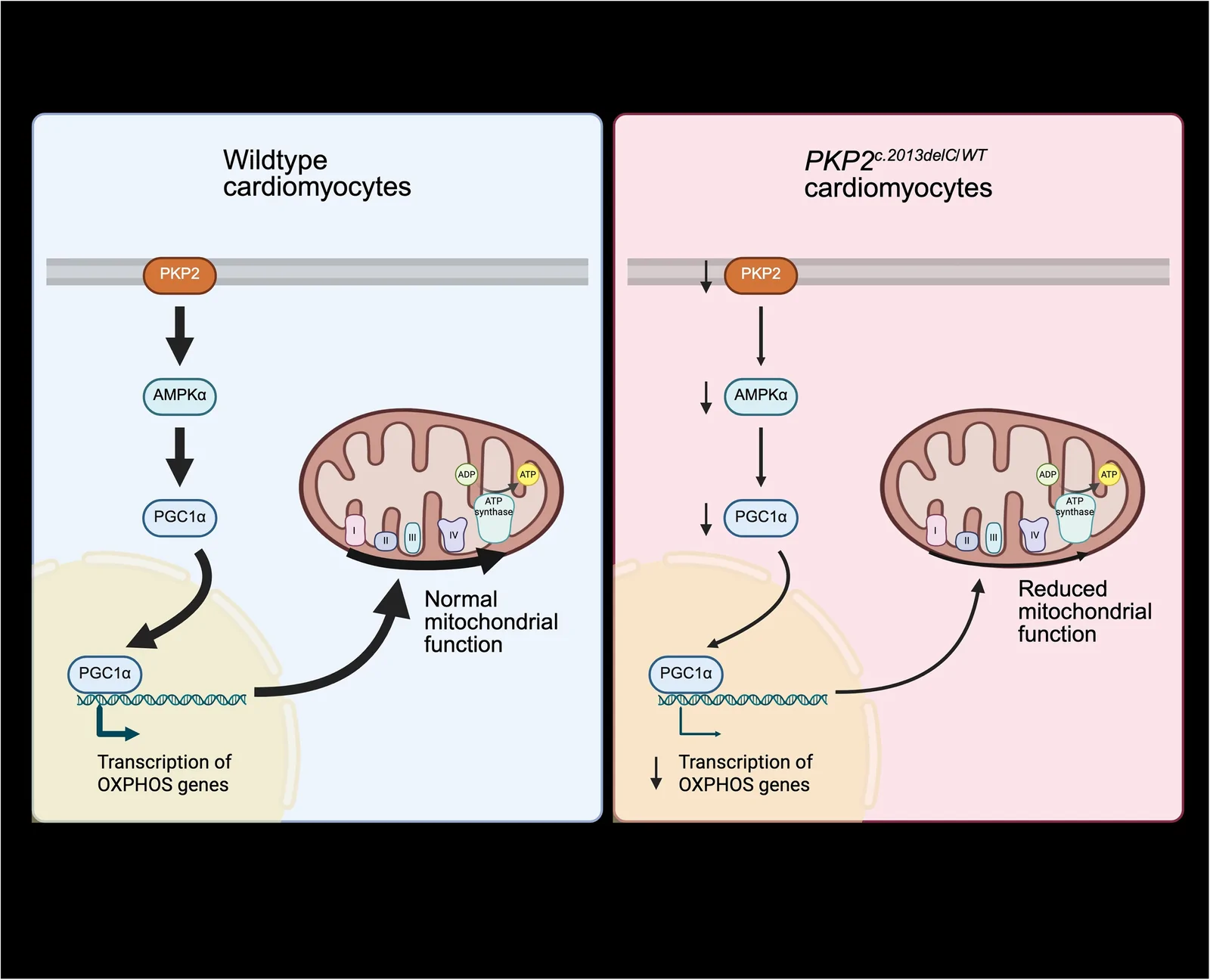

**Time of primary review: 26 days**

## 1. Introduction

Arrhythmogenic cardiomyopathy (ACM) is a rare heart disease with a prevalence ranging from 1:2000 to 1:5000 and is one of the leading causes of sudden cardiac death in young adults and athletes.^1,2^ It is considered a disease of the desmosome, as in up to 50% of reported ACM cases patients have pathogenic variants in the desmosomal genes, plakophilin-2 (*PKP2*) being the most frequently mutated gene, followed by desmoplakin (*DSP*), desmoglein-2 (*DSG2*), desmocolin-2 (*DSC2*) and plakoglobin (*JUP*).^3,4^ Together these five components of the desmosome form an essential protein complex that provides mechanical strength to cells by linking neighboring cells together. They are also part of a bigger complex called the intercalated disc (ID) that includes ion channels, adherence junctions and gap junctions.^4,5^ Previous studies have demonstrated that the components of the desmosome interact closely together with other proteins within the ID, having a crucial role in electrical homeostasis and signal transduction.^6–10^

While the genetic basis of ACM is well studied, early diagnosis remains challenging as genetic predisposition alone does not always lead to development of ACM. There is a wide variability in phenotypic expression among patients.^11,12^ Some individuals experience sudden cardiac death as the first sign of the disease, while others may present with syncope, palpitations, or remain asymptomatic throughout life.^13,14^ In more advanced stages of ACM, cardiomyocyte loss and fibrofatty replacement become more pronounced, eventually leading to heart failure. Incomplete penetrance and variable phenotypic expression in ACM indicate that other contributing factors play a role in the manifestation of the disease.^14^

One of the environmental factors that has been associated with worse prognosis in ACM is exercise.^15,16^ Patients with ACM who participate in competitive sports are more likely to have an earlier onset of symptoms and an increased risk of ventricular arrhythmias or sudden cardiac death.^16,17^ During exercise, the heart relies on a continuous supply of ATP to sustain contractile activity. ATP is generated in the mitochondria through the oxidative phosphorylation (OXPHOS) pathway which consists of five multiprotein complexes (Complexes I–V) made up of proteins encoded by both the mitochondrial and the nuclear genome.^18^ Complexes I–IV are part of the electron transport chain (ETC) where electrons are transferred from one complex to the other. Apart from Complex II, the electron transfer within these complexes is used to build up a proton gradient to fuel the production of ATP by Complex V.^19^ Of all the ATP that is produced in the heart, 90% of ATP is used for contraction and relaxation and its storage is limited. Therefore, to sustain contractility during increased energy demand, such as exercise, the heart must rapidly adapt and increase its energy production.^18,20,21^ Although mitochondria play an important part in this process, their role in the pathogenesis of ACM remains poorly understood.

In this study, we investigated how *PKP2* deficiency affects mitochondrial pathways using hiPSC-derived cardiomyocytes carrying pathogenic *PKP2* variants and explanted myocardial tissue from ACM patients. *PKP2* mutant hiPSC-CMs displayed reduced AMPKα levels and decreased expression of *PPARGC1A* (*PGC1α*), a master regulator of mitochondrial homeostasis, leading to diminished OXPHOS gene expression and mitochondrial capacity. Inducing *PPARGC1A* expression partially increased OXPHOS gene expression and improved contractile performance. These findings identify altered PGC1α signaling as a central mechanism linking reduced *PKP2* expression to impaired mitochondrial function in ACM.

## 2. Methods

A more detailed description of the methods and materials used is provided in the Supplementary material online.

### 2.1 Experimental model and study participants

This study is part of the UCC-UNRAVEL biobank, a single-center research data platform that combines routine electronic health records enriched with deep phenotyping, genetic data, and standardized biobanking to facilitate research in (inherited) heart diseases (https://www.unravelrdp.nl/).^22^ The study was approved by the local institutional ethics review board (University Medical Center Utrecht, protocol UCC-UNRAVEL # 12-387) and conforms to the principles outlined in the Declaration of Helsinki. Informed consent was obtained or in certain cases waived by the ethics committee when obtaining informed consent was not possible due to death of the patient.

### 2.2 hiPSC culture and cardiomyocyte differentiation

hiPSCs were maintained on Geltrex™ LDEV-Free, hESC-Qualified, Reduced Growth Factor Basement Membrane Matrix-coated wells (Gibco) in Essential 8™ Medium (Gibco). Directed differentiation to cardiomyocytes was performed following a small-molecule Wnt-modulation protocol (details can be found in the Supplementary material online).

### 2.3 Quantitative PCR

Total RNA was isolated from hiPSC-CMs using TRIzol™ (ThermoFisher) or RNeasy Mini Kit (Qiagen) according to the manufacturer’s protocol. Total RNA was reverse transcribed using the iScript™ cDNA Synthesis Kit (BioRad) and quantitative PCRs were performed on a Bio Rad CFX96 Real-Time PCR Detection System using the iQ SYBR Green Supermix kit (BioRad). The ΔΔCt method was used to analyze the data, and the sequences of the primers used for amplification are found in the Supplementary material online.

### 2.4 Immunoblotting

Proteins were extracted from hiPSC-CMs using RIPA buffer supplemented with protease and phosphatase inhibitors. 10–15 µg of protein were separated by SDS–PAGE and transferred to PVDF membranes. After blocking in 5% nonfat milk, membranes were incubated with primary antibodies overnight at 4°C and with HRP-conjugated secondary antibodies the following day. Signals were detected using Clarity™ ECL substrate and imaged with an Amersham Imager 680RGB. Band intensities were quantified using ImageJ. Antibody details and dilutions are provided in the Supplementary material online.

### 2.5 Knockdown experiments

Five to seven days after reseeding 3-week-old control hiPSC-CMs, cells were transfected with either 10 nM scramble or siRNA oligo duplexes targeting *PKP2*, *PPARGC1A*, or *DES* utilizing Lipofectamine™ 3000 Transfection Reagent (ThermoFisher) or Lipofectamine™ RNAiMAX (ThermoFisher) according to the manufacturers’ instructions.

### 2.6 Seahorse assay

hiPSC-CMs were seeded at 60 000 cells per well in 24-well Seahorse plates (Agilent). An XFe24 Analyzer (Agilent) was used to run the experiment with the following drug concentrations: 1 μM Oligomycin, 1 μM FCCP, Rotenone, and Antimycin A 2.5 μM. For normalization, the CyQUANT™ Cell Proliferation Assay (ThermoFisher) was used according to the manufacture’s protocol, and the data were analyzed using the Seahorse Wave Desktop Software.

### 2.7 AICAR treatments

Five to seven days after reseeding, cells were treated with 500 µM AICAR (Merck) for 15 min to isolate protein and for 24 h to isolate RNA. Protein and RNA were isolated as described above.

### 2.8 Overexpression experiments

Five to seven days after reseeding, cells were infected with either PPAGRC1A or EGFP cloned into the pLVX-IRES-Hygro vector backbone (Clontech). After 7 days, RNA and protein samples were collected (as described above). The viral particles used were harvested 48 h after the transfection of 7 × 10^6^ HEK293X cells with 11.9 μg pLVX-PPARGC1A gene or 10 µg pLVX-EGFP together with 9 μg psPAX2 and 3 μg pMD2G.

### 2.9 Immunocytochemistry

Human-explanted heart sections underwent antigen retrieval by boiling in 10 mM sodium citrate (pH 6.0) for 20 min, followed by cooling and Phosphate Buffered Saline (PBS) washes. Sections were blocked for 1 h in 0.5% Triton X-100 and 0.5% Bovine Serum Albumin in PBS and then incubated overnight at 4°C with primary antibodies diluted in blocking buffer. After washing, samples were incubated for 1 h with fluorescent secondary antibodies and DAPI. After three washing steps, the sections were mounted with Mowiol. Images were acquired using an Olympus VS200 Research Slide Scanner.

hiPSC-CMs were cultured on Geltrex-coated coverslips and fixed with 4% paraformaldehyde for 30 min at room temperature. After permeabilization with 0.1% Triton X-100, cells were blocked with 4% goat serum and incubated overnight at 4°C with primary antibodies diluted in blocking solution. Following washes, cells were incubated with fluorescent secondary antibodies and DAPI for 1 h at room temperature. After washing the coverslips, they were mounted with Mowiol. Images were acquired using a Leica TCS SPE confocal microscope. Antibody information is provided in the Supplementary material online.

### 2.10 Engineered human myocardium generation and contraction analysis

For generating engineered human myocardium (EHM), the protocol published by Tiburcy *et al.* was followed.^23^ Contraction measurements were performed using video-optic recordings of EHM-mediated pole bending in a myrPlate-TM5 culture format at 37°C. The spontaneously contracting EHM was recorded for at least 2 min at 50 fps at the indicated time points in a myrImager prototype (myriamed GmbH). Percent pole bending is reported as a surrogate for force of contraction.

### 2.11 Bulk RNA sequencing

The bulk RNA-sequencing dataset of *PKP2^c.2013delC/WT^* and its isogenic control *PKP2^WT/WT^* hiPSC-CMs in the GEO-database under accession GSE160289 has been used to analyze differential expression and KEGG pathway analysis.

### 2.12 Single-cell RNA sequencing and analysis

hiPSC-CMs were dissociated, single-cell sorted into 384-well plates (FACSJazz™ Cell Sorter, BD Biosciences) and processed for library preparation by Single Cell Discoveries using an adapted SORT-seq protocol.^24^ Sequencing data (available under the GEO accession number GSE298539) were analyzed in Seurat (v5.1.0) following standard workflows. Full details are provided in the Supplementary material online.

### 2.13 GTEx data analysis

Adult bulk RNA-sequencing data specific for left ventricular heart tissue, including the de-identified metadata, were obtained from the GTEx portal (V8 release) at gtexportal.org on 31 July 2024. Expression data were downloaded as transcripts per million and filtered for the target gene of interest (*PKP2* or *PPARGC1A*) for further downstream applications. A basic R script (build 4.2) was written to perform rank and correlation analyses for said genes.

### 2.14 Quantification and statistical analysis

Data are presented as the mean ± standard error of the mean and analyzed using GraphPad Prism 10. First, outliers were identified using the Robust regression and Outlier removal method (*Q* = 1%) and removed if present. Then, the data points were checked for normality using the D’Agostino and Pearson test and Anderson–Darling test. If normally distributed, the results were assessed for significance using a two-tailed unpaired Student’s *t* test when comparing two groups or an ordinary one-way analysis of variance (ANOVA) when comparing more than two groups. If the data are not normally distributed, two-tailed Mann–Whitney test was used to assess significance when comparing two groups and Kruskal–Wallis test when comparing more than two groups.

## 3. Results

### 3.1 Patient-derived *PKP2* mutant cardiomyocytes show decreased OXPHOS expression and mitochondrial function

In an effort to define cardiomyocyte intrinsic mechanisms underlying ACM, we used a human induced pluripotent stem cell line derived from a patient harboring the pathogenic *PKP2 c.2013delC* variant. Previous studies have shown that the *PKP2 c.2013delC* variant results in a premature stop codon causing haploinsufficiency in PKP2 and decreased desmosomal protein expression in *PKP2^c.2013delC/WT^* hiPSC-CMs compared to its isogenic control.^25–27^ We interrogated gene expression differences between *PKP2^c.2013delC/WT^* and its isogenic control *PKP2^WT/WT^* hiPSC-CMs by using bulk RNA-sequencing data and were able to show a clear separation when projected on a PCA plot, indicating significant differences in gene expression between *PKP2^c.2013delC/WT^* hiPSC-CMs and its isogenic control (*Figure 1A*). We identified 3564 up- and 3591 downregulated genes in the mutant cells compared to control (Log2FoldChange > 0.1 and <−0.1, adjusted *P*-value (*P*_adj_) < 0.05) (*Figure 1B*). Pathway analysis of the significantly differentially expressed genes indicated an enrichment for genes related to Wnt– and PI3K–Akt signaling; both pathways have been previously described as being implicated in the pathogenesis of ACM and other cardiomyopathies.^10,28,29^ However, among the suppressed pathways, we identified the OXPHOS pathway as being significantly suppressed in *PKP2* mutant cells compared to control (*Figure 1C*). Most of the genes related to OXPHOS that were downregulated in the *PKP2^c.2013delC/WT^* hiPSC-CMs, were encoded in the nuclear genome and not confined to a specific complex (see Supplementary material online, *Table S1*). The decreased expression of OXPHOS associated genes that were identified by the RNA sequencing (*NDUFA4*, *NDUFB2*, *NDUFA13*, and *NDUFS6* of Complex I, *UQCRQ* of Complex III, and *COX6A1*, *COX6B1*, and *COX17* of Complex IV) could be validated in two additional differentiations of cardiomyocytes (*Figure 1D*). The decline in mRNA expression also corresponded to reduced protein levels of genes involved in OXPHOS in the *PKP2* mutant hiPSC-CMs compared to the control (*Figure 1E* and *F*).

**Figure 1:**
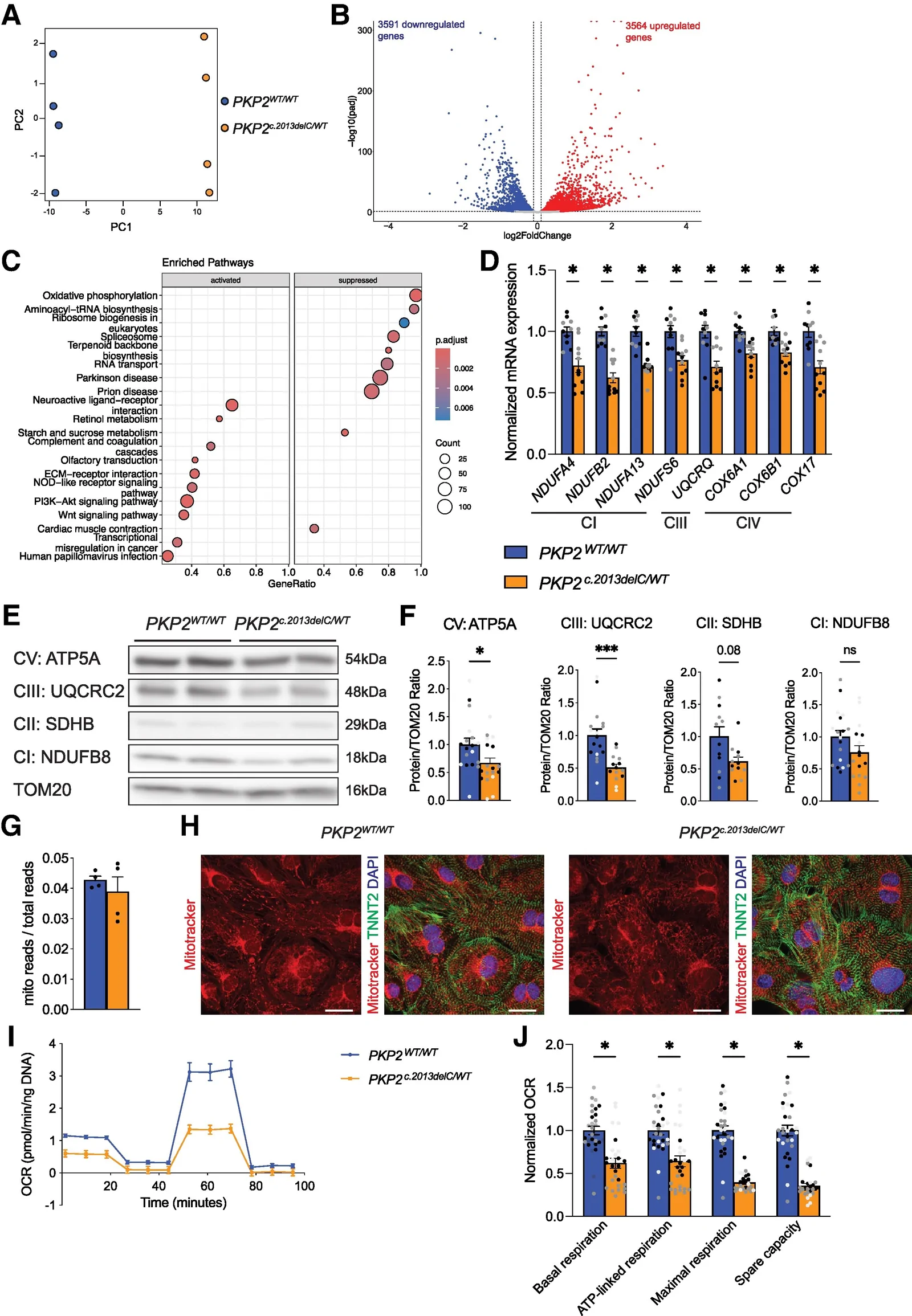
Expression of OXPHOS-related genes and mitochondrial function in *PKP2^c.2013delC/WT^* hiPSC-CM. (*A*) Principal component analysis of *PKP2^c.2013delC/WT^* and its isogenic control *PKP2^WT/WT^* hiPSC-CMs of the bulk RNA sequencing. (*B*) Volcano plot showing the up- and downregulated genes in *PKP2^c.2013delC/WT^* compared to *PKP2^WT/WT^* hiPSC-CMs (log2FoldChange > 0.1 and <−0.1, *P*_adj_ < 0.05). (*C*) KEGG-enriched pathway analysis of the dysregulated genes in *PKP2^c.2013delC/WT^* hiPSC-CMs compared to the isogenic control. (*D*) Validation of decreased gene expression of OXPHOS-related genes in *PKP2* mutant and wildtype hiPSC-CMs (CI: Complex I, CIII: Complex III, CIV: Complex IV). Data plotted as mean. The dots represent technical replicates, while the color of each dot indicates the different batches of differentiation (two independent experiments; four to six technical replicates). Significance was assessed by a two-tailed unpaired Student’s *t* test (*P < 0.05). (*E*) Representative Western blot of OXPHOS proteins from different complexes (CI: Complex I, CII: Complex II, CIII: Complex III, CV: Complex V). (*F*) Quantification of OXPHOS protein levels normalized to TOM20. Data plotted as mean. The dots represent technical replicates, while the color of each dot indicates the different batches of differentiation (two to three independent experiments; twp to six technical replicates). Significance was assessed by a two-tailed unpaired Student’s *t* test or two-tailed Mann–Whitney test when data were not normally distributed (*P < 0.05, ***P < 0.001, ns, not significant). (*G*) Quantification of mitochondrial reads from the bulk RNA sequencing. (*H*) Immunofluorescence images of *PKP2^c.2013delC/WT^* and *PKP2^WT/WT^* hiPSC-CMs stained with Mitotracker in red, cardiac troponin T (TNNT2) in green and DAPI in blue. Scale bar: 20 µm. (*I*) Oxygen consumption rate (OCR) of *PKP2^c.2013delC/WT^* and *PKP2^WT/WT^* hiPSC-CMs during the Seahorse Mito Stress test. (*J*) Quantification of basal, ATP-linked, maximal respiration, and spare capacity of the Mito Stress test. Data plotted as mean. The dots represent technical replicates, while the color of each dot indicates the different batches of differentiation (three independent experiments; eight to ten technical replicates). Significance was assessed by a two-tailed unpaired Student’s *t* test or two-tailed Mann–Whitney test when data were not normally distributed (*P < 0.05).

To explore whether the decline in expression of genes associated with OXPHOS was due to a reduction in mitochondrial content, we investigated the number of mitochondrial reads in the bulk RNA-sequencing data. In addition, we stained the *PKP2^c.2013delC/WT^* and *PKP2^WT/WT^* hiPSC-CMs with the MitoTracker™ dye. Both assays indicated no difference in mitochondrial content between the mutant and control cells (*Figure 1G* and *H*). This suggests that the suppression of the OXPHOS pathway in the *PKP2^c.2013delC/WT^* cells is regulated at the transcriptional level rather than it being a consequence of a change in mitochondrial content.

Next, we investigated whether the decrease in expression of OXPHOS components corresponded to a decline in mitochondrial function in the *PKP2* mutant cells and performed a Seahorse assay. This indicated that the *PKP2^c.2013delC/WT^* hiPSC-CMs showed lower basal, ATP-linked and maximal respiration, which consequently led to decreased spare capacity, a measure for the flexibility of a cell to respond to increased energy demand or stress (*Figure 1I* and *J*). To interrogate whether these observations reflected a common mechanism, we performed comparable analyses on hiPSC-CMs harboring the *PKP2 c.1849C>T* variant. Similar to the *PKP2 c.2013delC* variant, the *PKP2 c.1849C>T* variant is a nonsense variant that leads to a premature stop codon and causes PKP2 haploinsufficiency (see Supplementary material online, *Figure S1A*–*D*).^30^ As previously shown, decreased levels of PKP2 led to a decline in other desmosomal proteins DSP and JUP while leaving mRNA expression of the desmosomal genes *DSP*, *JUP*, *DSC2*, and *DSG*2 mostly unaffected (see Supplementary material online, *Figure S1A*–*D*).^26,27^ In addition, we found a significant reduction in the expression of OXPHOS-related genes in the *PKP2^c.1849C>T/WT^* hiPSC-CMs compared to its isogenic control (see Supplementary material online, *Figure S1E*–*G*), which led to lower spare capacity in the mutant cells as measured by the Seahorse assay (see Supplementary material online, *Figure S1H* and *I*).

Together, these data demonstrate that hiPSC-CMs harboring pathogenic *PKP2* variants leading to PKP2 haploinsufficiency have decreased expression of genes involved in OXPHOS that corresponds to a lowering in mitochondrial function.

### 3.2 Expression of OXPHOS-related genes and mitochondrial function are directly regulated by the level of PKP2

To further investigate the correlation between the level of PKP2 and mitochondrial function, we aimed to dose-dependently inhibit *PKP2* in healthy control hiPSC-CMs (*Figure 2A*). To do so, control cells were treated with increasing concentrations of an siRNA targeting *PKP2* for 3–5 days after which molecular and functional analyses were performed. Real-time PCR analysis indicated a dose-dependent reduction of *PKP2* mRNA levels (*Figure 2B*), which also translated into decreased PKP2 protein levels (*Figure 2C–E*). In addition, similar to the hiPSC-CMs harboring a *PKP2* pathogenic variant, JUP expression was decreased in a dose-dependent manner after 4 days of siRNA treatment (*Figure 2D* and *E*). DSP expression was only significantly reduced 5 days after the start of the siRNA treatment, suggesting that loss of PKP2 first affects JUP expression before leading to a decline in DSP (see Supplementary material online, *Figure S2A* and *B*).

**Figure 2:**
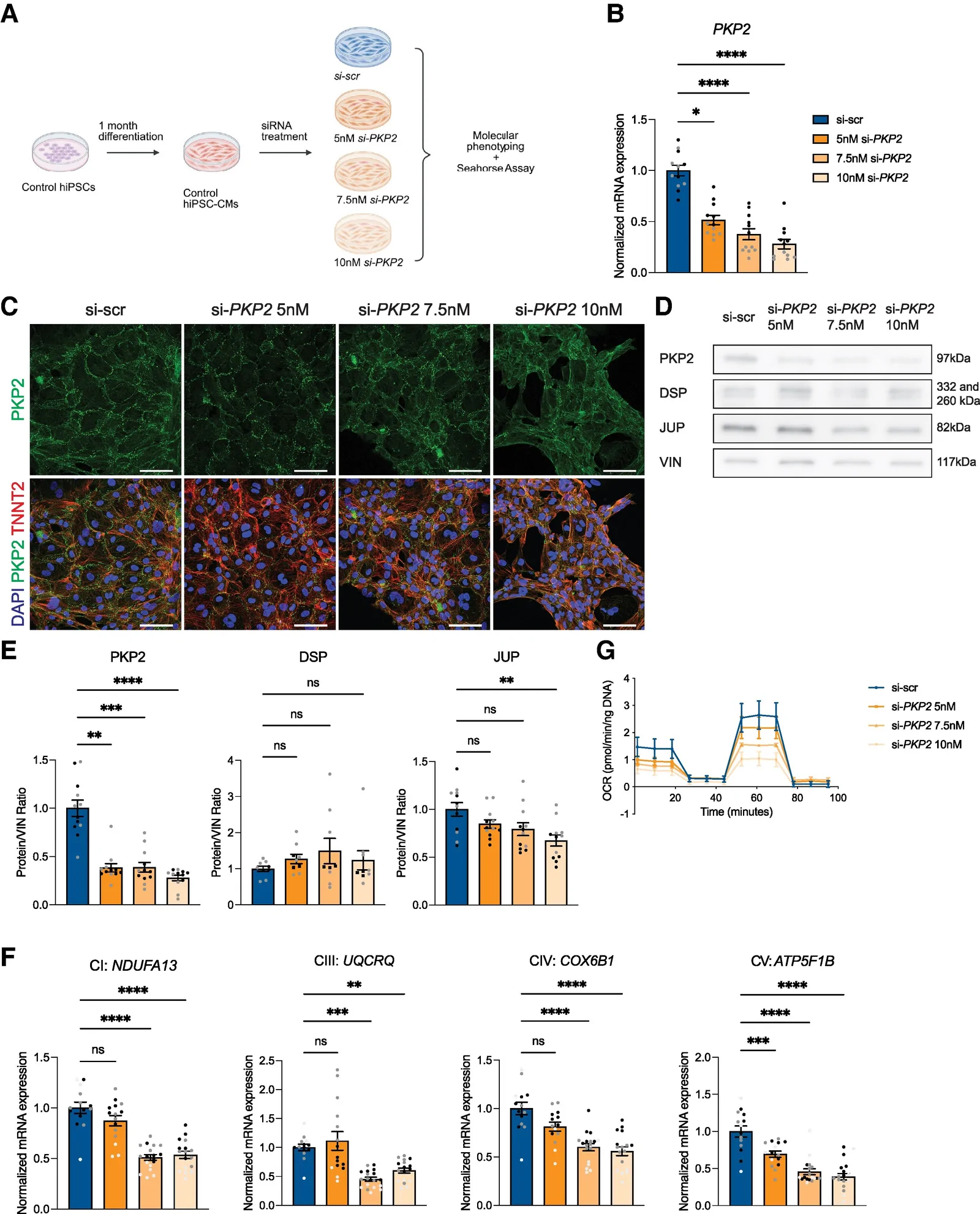
Phenotyping of control hiPSC-CMs after dose-dependent knockdown of *PKP2* using siRNA. (*A*) Schematic representation of the knockdown experiment using control hiPSC-CMs and siRNA targeting *PKP2*. Created in Biorender (https://BioRender.com/w068a4a). (*B*) *PKP2* mRNA expression of control hiPSC-CMs treated for 3 days with different doses of si-*PKP2*. Data plotted as mean. The dots represent technical replicates, while the color of each dot indicates the different batches of differentiation (two independent experiments; six technical replicates). Significance was assessed by an Kruskal–Wallis test followed by Dunn’s multiple comparisons test (*P < 0.05, ****P < 0.0001). (*C*) Immunofluorescence images of control hiPSC-CMs after 4 days of si-*PKP2* treatment stained with PKP2 in green, cardiac troponin T (TNNT2) in red, and DAPI in blue. Scale bar: 60 µm. (*D*) Representative Western blot of the desmosomal proteins PKP2, DSP and JUP after 4 days of si-*PKP2* treatment. (*E*) Quantification of the desmosomal protein levels normalized to VIN. Data plotted as mean. The dots represent technical replicates, while the color of each dot indicates the different batches of differentiation (two independent experiments; three to six technical replicates). Significance was assessed by an ordinary one-way ANOVA or Kruskal–Wallis test when data were not normally distributed followed by Dunnett’s or Dunn’s multiple comparisons test respectively (**P < 0.01, ***P < 0.001, ****P < 0.0001, ns, not significant). (*F*) mRNA expression of OXPHOS-related genes from different complexes after 5 days of si-*PKP2* treatment (CI: Complex I, CIII: Complex III, CIV: Complex IV, CV: Complex V). Data plotted as mean. The dots represent technical replicates, while the color of each dot indicates the different batches of differentiation (three independent experiments; three to six technical replicates). Significance was assessed by an ordinary one-way ANOVA or Kruskal–Wallis test when data were not normally distributed followed by Dunnett’s or Dunn’s multiple comparisons test, respectively (**P < 0.01, ***P < 0.001, ****P < 0.0001, ns, not significant). (*G*) Oxygen consumption rate (OCR) of control hiPSC-CMs after 5 days of si-*PKP2* treatment.

After confirming the reduction of PKP2 expression, we studied the expression of OXPHOS associated genes in the si-*PKP2* treated hiPSC-CMs. Most of the genes that showed a decreased expression in the *PKP2^c.2013delC/WT^* hiPSC-CMs were also reduced after knockdown of *PKP2* in a dose-dependent manner (*Figure 2F* and Supplementary material online, *Figure S2C*). In line with these results, mitochondrial function was decreased in healthy hiPSC-CMs upon PKP2 inhibition in a dose-dependent manner (*Figure 2G*). These data indicate that loss of PKP2 directly affects expression of genes involved in OXPHOS and consequently influences mitochondrial function.

Given the known interactions between desmosomes, cytoskeletal proteins, and mitochondria, we investigated whether *PKP2* deficiency affects cytoskeletal organization and whether this contributes to altered OXPHOS gene expression. We first examined desmin (DES), an intermediate filament protein previously linked to ACM.^31^ DES expression was significantly reduced in *PKP2^c.2013delC/WT^* hiPSC-CMs compared to isogenic controls and appeared to be *PKP2* dose-dependent (see Supplementary material online, *Figure S3A*–*C*). To assess whether DES influences gene expression in the OXPHOS pathway, we silenced *DES* expression in control hiPSC-CMs using siRNA. Despite efficient knockdown, we observed no significant changes in the expression of OXPHOS-related genes, except for a modest reduction in *UQCRQ* (see Supplementary material online, *Figure S3D*). To evaluate whether other cytoskeletal elements are altered in *PKP2* mutant cells, we stained *PKP2^c.2013delC/WT^* and *PKP2^WT/WT^* hiPSC-CMs for α-tubulin (microtubules) and phalloidin (F-actin) (see Supplementary material online, *Figure S3E* and *F*). However, no obvious differences in their organization or abundance were detected. These findings suggest that although PKP2 deficiency alters DES expression, this is unlikely to be a primary driver of the decreased expression of OXPHOS genes we observed in *PKP2^c.2013delC/WT^* hiPSC-CMs.

### 3.3 *PKP2* expression is correlated to *PPARGC1A* expression in hiPSC-CMs

Since our data indicated PKP2 levels to direct transcriptional changes in cardiomyocytes, we set out to gain more in-depth insights into the genes correlated to the level of PKP2. To this end, we performed single-cell RNA sequencing on the *PKP2^c.2013delC/WT^* and *PKP2^WT/WT^* hiPSC-CMs. In doing so, we were able to identify nine different cardiomyocyte clusters that separated mostly based on the genotype of the cells, suggesting that the mutant and control cardiomyocytes have very distinct molecular phenotypes (*Figure 3A* and *B* and Supplementary material online, *Figure S4A*). In line with previous findings, the expression of desmosomal genes did not differ between different clusters or cell lines except for *PKP2* (*Figure 3C* and Supplementary material online, *Figure S4B* and *C*).^26,27^ Next, we checked whether the downregulated genes in *Figure 1* could be corroborated in the single-cell dataset. *NDUFA4*, *UQCRQ*, *COX6B1*, and *ATP5F1* were downregulated in the *PKP2^c.2013delC/WT^* hiPSC-CMs compared to the *PKP2^WT/WT^* hiPSC-CMs (*Figure 3D*). Furthermore, gene set enrichment analysis between *PKP2* mutant and control cells highlighted OXPHOS pathways to be suppressed in the mutant cells (see Supplementary material online, *Figure S4D*), indicating the reproducibility of our reported findings.

**Figure 3:**
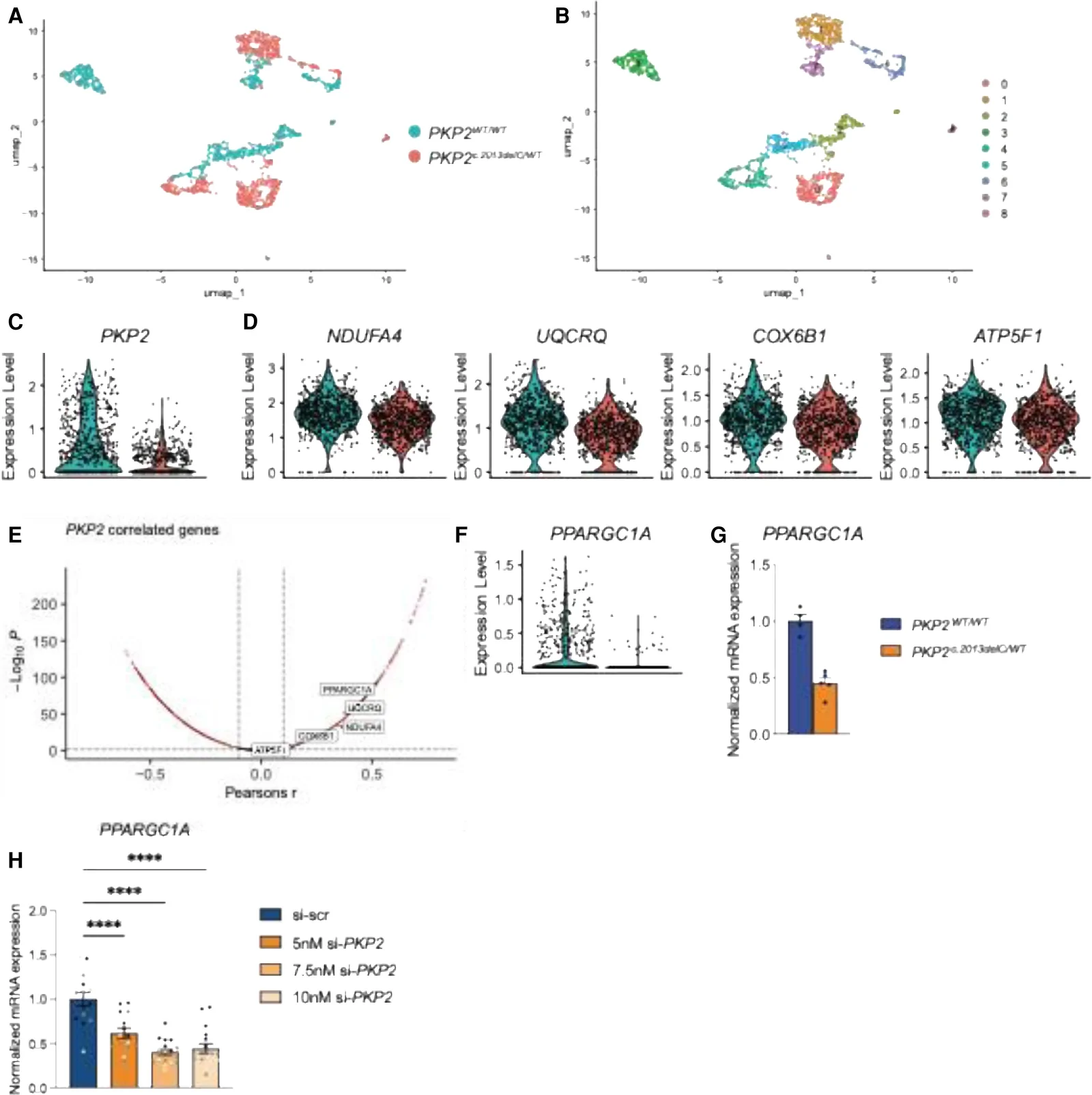
Single-cell RNA-sequencing analysis of *PKP2^c.2013delC/WT^* and *PKP2^WT/WT^* hiPSC-CMs. (*A* and *B*) UMAP displaying the cells used for single-cell RNA-sequencing analysis based on genotype and clusters identified by Seurat. (*C* and *D*) Violin plot showing expression of *PKP2* and genes related to OXPHOS in *PKP2^c.2013delC/WT^* and *PKP2^WT/WT^* hiPSC-CMs. (*E*) Analysis of *PKP2*-correlated genes depicting genes that are involved in OXPHOS. (*F*) Violin plot showing *PPARGC1A* expression in *PKP2^c.2013delC/WT^* and *PKP2^WT/WT^* hiPSC-CMs. (*G*) Validation of *PPARGC1A* mRNA expression in another batch of differentiation (four to five technical replicates). (*H*) *PPARGC1A* mRNA expression in control hiPSC-CMs treated with different concentrations of si-*PKP2* after 5 days. Data plotted as mean. The dots represent technical replicates, while the color of each dot indicates the different batches of differentiation (three independent experiments; three to six technical replicates). Significance was assessed by an ordinary one-way ANOVA followed by Dunnett’s multiple comparisons test (****P < 0.0001).

Single-cell RNA sequencing not only allows for determining intercellular gene expression differences, but also provides a mean for examining gene expression correlations at the single-cell level.^32^ As we previously showed that OXPHOS-related gene expression is affected by loss of *PKP2*, we next used the single-cell RNA-sequencing data to look at *PKP2* correlated genes. Among the negatively correlated genes, we mostly found genes related to the ribosome (*RPL15*, *RPS5*, and *RPL27A*) but also found translation elongator factors (*EEF1A1* and *EEF*2) as well as the cytoskeleton gene *ACTG1* (see Supplementary material online, *Figure S4E*). Among the positively correlated genes, genes related to sarcomere (*ACTC1*, *MYBPC3*, and *TTN*) and glycolysis (*PFKP*, *ALDOA*, and *PKM*) but also related to OXPHOS (*NDUFA4*, *UQCRQ*, *COX6B1*, and *ATP5F1*) were observed (see Supplementary material online, *Figure S4E* and *Figure 3E*).

To validate the correlation of *PKP2* and glycolytic genes, we checked the expression of *PFKP*, *ALDOA*, and *PKM* in our dose-dependent knockdown experiment of *PKP2*. They were significantly downregulated in a dose-dependent manner (see Supplementary material online, *Figure S4F*). In addition, genes related to fatty acid metabolism such as *CPT1A* and *FABP3* were reduced after si-*PKP2* suggesting impaired fatty acid import and oxidation upon PKP2 loss (see Supplementary material online, *Figure S4G*). These transcriptional observations suggest that *PKP2* deficiency also induces changes in glycolytic and fatty acid pathways.

In search for transcriptional activators that might be responsible for the regulation of the OXPHOS genes, we identified *PPARGC1A*, which encodes for the PGC1α protein, to be positively correlated with *PKP2* (*Figure 3E*). PGC1α is a transcription factor known as a key regulator of mitochondrial genes that are expressed from the nuclear DNA.^33–35^ As previous studies have shown that *PPARGC1A* also influences the expression of OXPHOS genes in mouse hearts, we set out to explore whether a decline in PGC1α might be the underlying cause for the mitochondrial phenotype we observed in the *PKP2* mutant cardiomyocytes.^36^

Single-cell RNA-sequencing data indicated a decrease in *PPARGC1A* expression in the *PKP2^c.2013delC/WT^* hiPSC-CMs compared to its control (*Figure 3F*), which was confirmed in an additional differentiation and by the bulk RNA-sequencing data (*Figure 3G* and Supplementary material online, *Table S1*). The correlation between *PPARGC1A* expression and the loss in PKP2 was further confirmed by the dose-dependent decrease in *PPARGC1A* expression after knocking down *PKP2* in healthy hiPSC-CMs (*Figure 3H*).

Next, to study whether the decreased expression of PGC1α is causing the downregulation of OXPHOS-related genes in hiPSC-CMs, we treated healthy hiPSC-CMs with an siRNA targeting *PPARGC1A*. After confirming the knockdown of *PPARGC1A*, we looked at the expression of several OXPHOS genes (see Supplementary material online, *Figure S5A* and *B*). Reminiscent of *PKP2* mutant hiPSC-CMs, expression of OXPHOS-related genes, such as *NDUFA4*, *UQCRQ*, *COX6B1*, and *ATP5PF*, were decreased in cells treated with siRNA against *PPARGC1A*. Moreover, other genes of the OXPHOS complexes were also downregulated, suggesting that PGC1α is an important regulator of genes involved in OXPHOS that are encoded by the nuclear genome (see Supplementary material online, *Figure S5C*–*F*). Together, these findings suggest that the expression of the transcription factor *PPARGC1A* is affected by the level of *PKP2* and regulates OXPHOS-related gene expression in hiPSC-CMs.

### 3.4 Human explanted hearts harboring different *PKP2* variants show differences in localization of PGC1α and expression of OXPHOS components compared to control hearts

Because our data pointed to a link between PKP2 haploinsufficiency and a decrease in the expression of *PPARGC1A* and OXPHOS components in human *in vitro* models of ACM, we wanted to further investigate the relevance of our findings in human adult hearts. To do so, we made use of the publicly available GTEx database containing bulk RNA-sequencing samples from left ventricular non-diseased human hearts derived from more than 400 individuals. Natural genetic variation of *PKP2* or *PPARGC1A* levels (*Figure 4A* and see Supplementary material online, *Figure S6A*) within this population cohort permitted us to correlate expression levels across individuals to infer potential gene function. In line with our results of the hiPSC-CMs, *PKP2* expression was positively correlated to OXPHOS-related genes, such as *NDUFA4*, *UQCRQ*, *COX6B1*, and *ATP5F1*, and *PPARGC1A* in the GTEx dataset (*Figure 4B* and *C*). The same was observed when looking at *PPARGC1A* correlated genes (see Supplementary material online, *Figure S6B*). Furthermore, sarcomeric (*ACTC1*, *MYBPC3*, *TTN*) and glycolytic genes (*PFKP*, *ALDOA*, *PKM*) that were identified in our single-cell RNA-sequencing dataset of hiPSC-CMs also showed high positive correlation to *PKP2* expression in adult non-diseased hearts (see Supplementary material online, *Figure S6C*). These results indicate that we can validate the findings in our single-cell RNA sequencing of our *PKP2^c.2013delC/WT^* hiPSC-CMs in human adult hearts.

**Figure 4:**
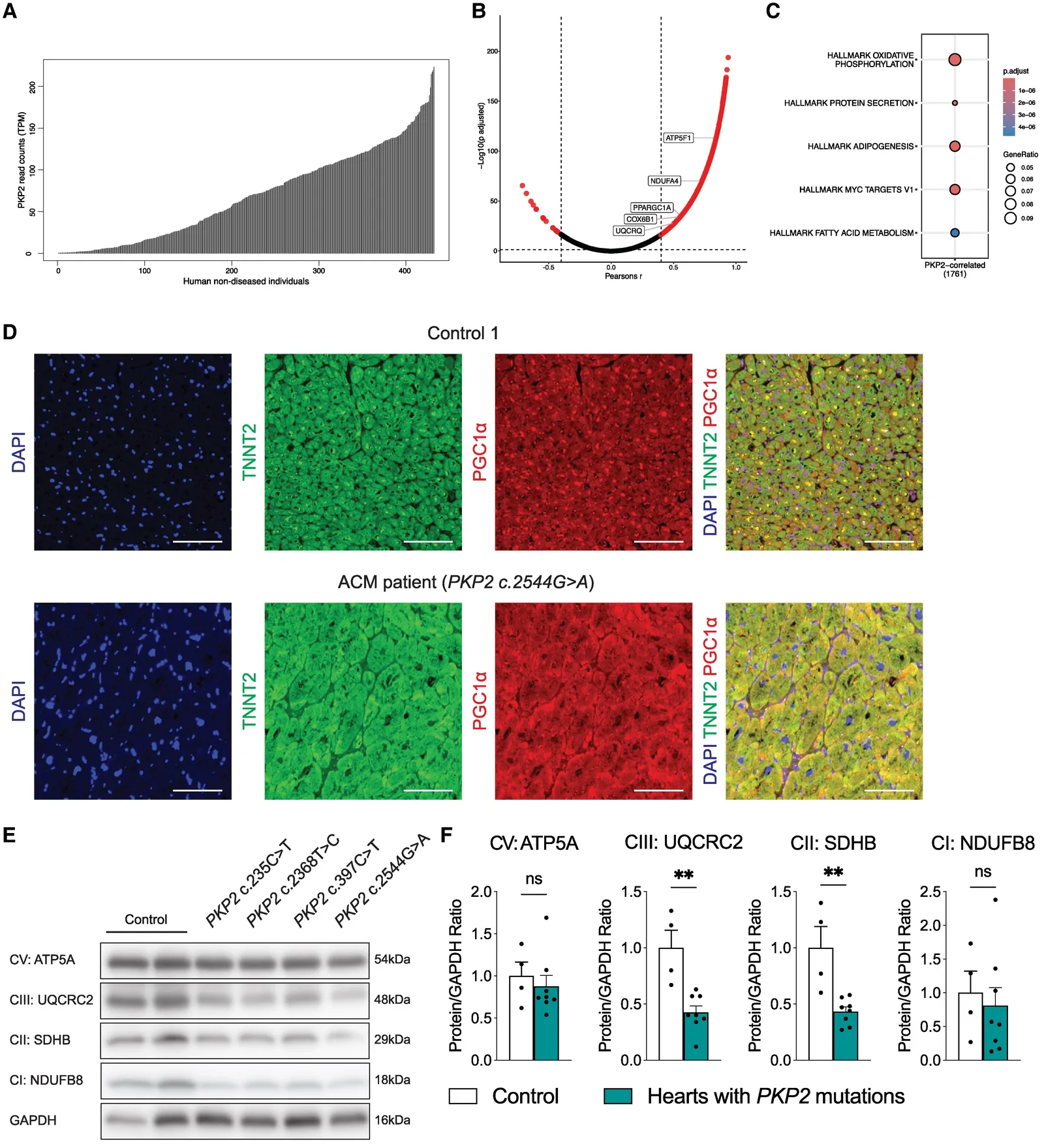
Expression of PGC1α and OXPHOS-related proteins in human explanted hearts from ACM patients with a *PKP2* variant. (*A*) Graph showing the read counts of *PKP2* across the non-diseased human heart samples taken from the GTEx dataset. (*B*) Graph showing the correlated genes related to OXPHOS with *PKP2*. (*C*) Gene enrichment analysis of positively correlated genes with *PKP2* using the Hallmarks dataset that is part of the Molecular Signatures Database (MSigDB). (*D*) Immunofluorescence images of left ventricular tissue collected from an explanted control and ACM heart stained with PGC1α in red, cardiac troponin T (TNNT2) in green and DAPI in blue. Scale bar: 100 μm. (*E*) Representative Western blot of OXPHOS proteins in two control human explanted hearts and hearts harboring different variants in *PKP2* (CI: Complex I, CII: Complex II, CIII: Complex III, CV: Complex V). (*F*) Quantification of the OXPHOS protein levels normalized to GAPDH combining protein samples from the right and left ventricle with and without a *PKP2* variant. Significance was assessed by a two-tailed unpaired Student’s *t* test or two-tailed Mann–Whitney test when data were not normally distributed (**P < 0.01, ns, not significant).

Based on the correlation between *PPARGC1A* and *PKP2* expression in our hiPSC-CMs and human adult hearts, we next wanted to assess how PGC1α is expressed in explanted hearts from ACM patients. We stained left ventricular heart tissue from two non-diseased individuals and four ACM patients with different *PKP2* variants for cardiac troponin T (TNNT2) and PGC1α (*Figure 4D* and Supplementary material online, *Figure S6D*). All ACM patients who were included in this study initially presented with ACM symptoms that developed into bi-ventricular heart failure at the time of heart transplantation and showed decreased desmosomal protein expression, similar to our patient-derived hiPSC-CMs (see Supplementary material online, *Table S2*).^27^ We observed that PGC1α is less clearly localized in the nucleus in the *PKP2* mutant hearts compared to the control hearts suggesting that its function as a transcription factor is dysregulated in the hearts of ACM patients. To investigate whether this affects the expression of OXPHOS components, proteins from left and right ventricular tissue of the non-diseased and *PKP2* mutant hearts were extracted. Decreased expression of SDHB and UQCRC2, proteins of Complexes II and III, respectively, was observed. For ATP5A and NDUFB8 there was a decreasing trend that was not significant (*Figure 4E* and *F*). Together, we show that the localization in human adult tissues of PGC1α is disturbed and the protein levels of OXPHOS-related components are also decreased in hearts harboring pathogenic *PKP2* variants, similar to the *PKP2* mutant hiPSC-CMs.

### 3.5 *PKP2^c.2013delC/WT^* hiPSC-CMs show decreased AMPKα levels and reduced ability to increase its activation following AICAR treatment

Based on our findings that *PPARGC1A* expression is altered in *PKP2* mutant cardiomyocytes and human explanted ACM hearts, and its downregulation contributes to reduced OXPHOS expression in *PKP2* mutant cardiomyocytes, we sought to identify upstream regulators that could link *PKP2* to the transcriptional control of *PPARGC1A*. Since PKP2 has been proposed to act as a scaffold for kinases, and PGC1α is regulated by kinases, we focused on AMP-activated protein kinase (AMPK), which has been shown to activate *PPARGC1A* and to localize to the ID in mouse cardiomyocytes.^37–39^ We observed significantly reduced total AMPKα expression in *PKP2^c.2013delC/WT^* hiPSC-CMs compared to its isogenic control, suggesting impaired energetic sensing in mutant cells (*Figure 5A* and *B*). To assess whether this impairs the response to exercise, we treated cells with AICAR, an AMP analog and pharmacological AMPK activator frequently used as an exercise mimetic.^40^ In *PKP2^WT/WT^* hiPSC-CMs, AICAR treatment led to an increase in AMPKα phosphorylation and upregulated *PPARGC1A* expression after 24 h. In contrast, *PKP2* mutant cells exhibited blunted AMPK phosphorylation and failed to induce *PPARGC1A* expression in response to AICAR (*Figure 5C–E*). These findings suggest that reduced AMPKα availability in *PKP2* mutant cardiomyocytes impairs the induction of PGC1α in response to energetic stress, potentially limiting their ability to adapt to increased energy demands during exercise.

**Figure 5:**
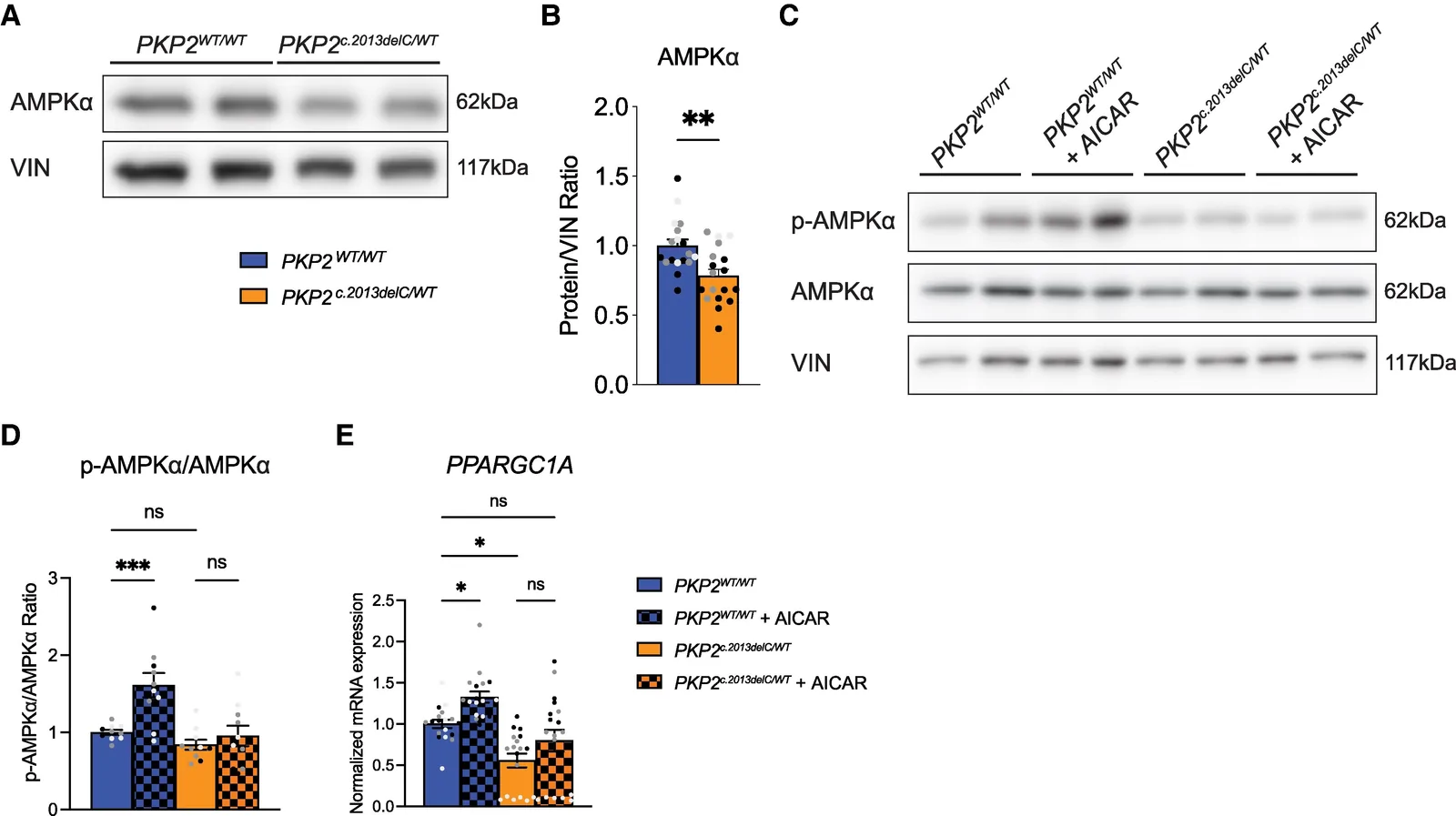
Expression of AMPKα and AICAR treatment in *PKP2^c.2013delC/WT^* and *PKP2^WT/WT^* hiPSC-CMs. (*A*) Representative Western blot of AMPKα protein levels. (*B*) Quantification of AMPKα levels normalized to VIN. Data plotted as mean. The dots represent technical replicates, while the color of each dot indicates the different batches of differentiation (three independent experiments; five to six technical replicates). Significance was assessed by a two-tailed Mann–Whitney test (**P < 0.01). (*C*) Representative Western blot of phosphorylated AMPKα and AMPKα after treatment of *PKP2^WT/WT^* and *PKP2^c.2013delC/WT^* hiPSC-CMs with 500 µM AICAR. (*D*) Quantification of the ratio of *P*-AMPKα and AMPKα after treatment with AICAR. Data plotted as mean. The dots represent technical replicates, while the color of each dot indicates the different batches of differentiation (three independent experiments; two to four technical replicates). Significance was assessed by an ordinary one-way ANOVA followed by Šidák’s multiple comparisons test (***P < 0.001, ns, not significant). (*E*) *PPARGC1A* expression of *PKP2^WT/WT^* and *PKP2^c.2013delC/WT^* hiPSC-CMs after treatment with 500 µM AICAR. Data plotted as mean. The dots represent technical replicates, while the color of each dot indicates the different batches of differentiation (three independent experiments; six technical replicates). Significance was assessed by a Kruskal–Wallis test followed by Dunn’s multiple comparisons test (*P < 0.05).

### 3.6 *PPARGC1A* overexpression increases OXPHOS-related gene expression and improves contractility in *PKP2^c.2013delC/WT^* hiPSC-CMs

The observation that induction of *PPARGC1A* is impaired in *PKP2^c.2013delC/WT^* hiPSC-CMs, likely because of decreased AMPKα protein levels, prompted us to examine whether overexpressing *PPARGC1A* directly could improve the expression of OXPHOS-related genes in the *PKP2^c.2013delC/WT^* hiPSC-CMs and lead to improvement in cardiomyocyte function. To test this hypothesis, we treated *PKP2^c.2013delC/WT^* and *PKP2^WT/WT^* hiPSC-CMs with lentivirus carrying either *GFP* or *PPARGC1A*. The level of *PPARGC1A* overexpression was similar in the control and *PKP2* mutant cells, which could be confirmed at both mRNA and protein level (*Figure 6A* and *B*). Expression of *NDUFA4*, *UQCRQ* and *COX5A* was significantly increased in both the *PKP2^c.2013delC/WT^* and *PKP2^WT/WT^* hiPSC-CMs after overexpressing *PPARGC1A* (*Figure 6C* and Supplementary material online, *Figure S7A*). *NDUFS8*, *UQCRFS1*, *ATP5MF*, and *ATP5FB1* showed a trend toward increased expression which was not significant and no change in *ATP5PF* expression was observed. *NDUFA13* and *COX6B1* only showed increased expression in the isogenic control after *PPARGC1A* induction (*Figure 6C* and Supplementary material online, *Figure S7A*).

**Figure 6:**
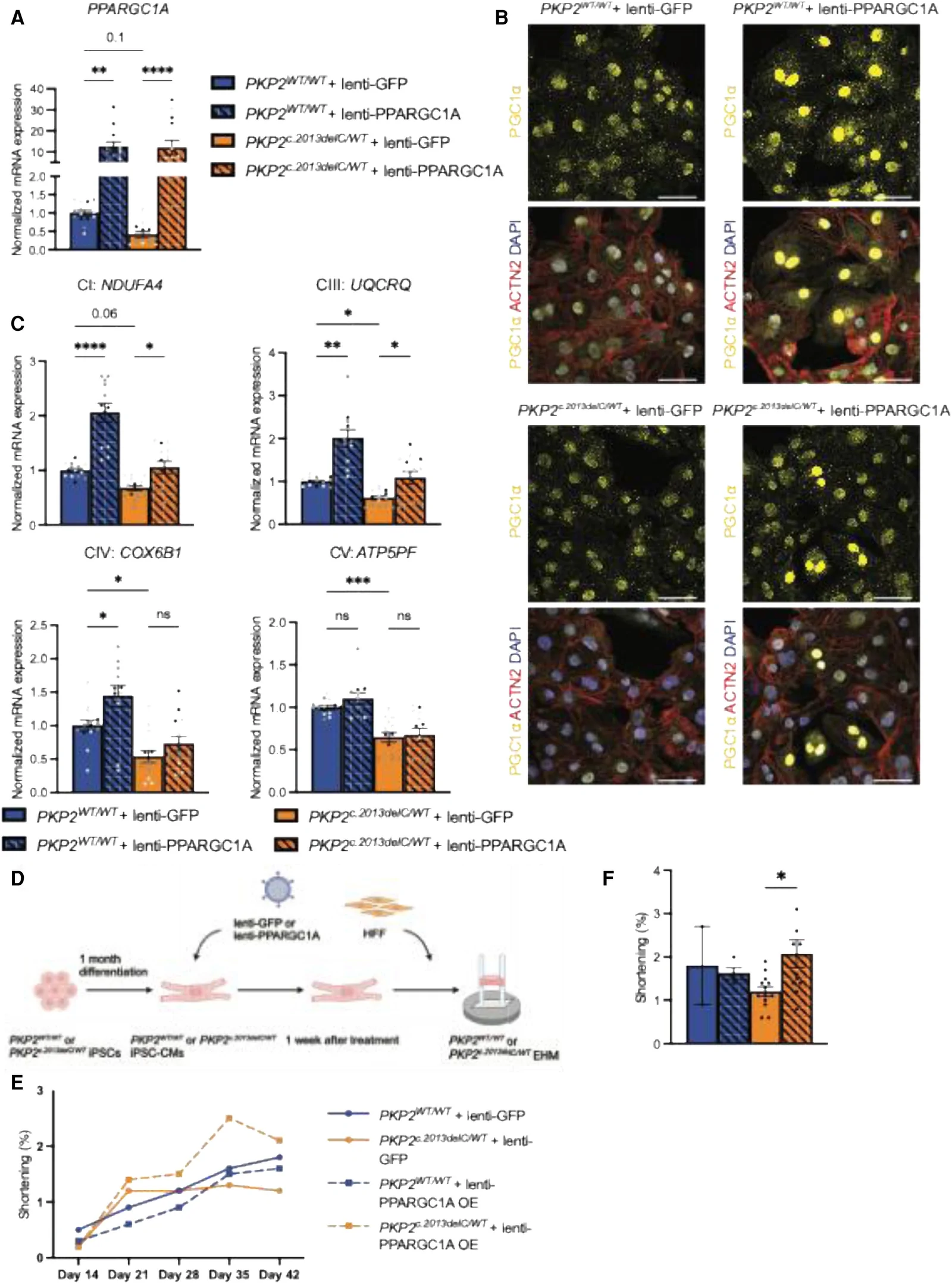
Expression of OXPHOS-related genes and contractility in *PKP2^c.2013delC/WT^* and *PKP2^WT/WT^* hiPSC-CMs after *PPARGC1A* overexpression. (*A*) *PPARGC1A* mRNA expression after lentivirus treatment in *PKP2^c.2013delC/WT^* and *PKP2^WT/WT^* hiPSC-CMs. Data plotted as mean. The dots represent technical replicates, while the color of each dot indicates the different batches of differentiation (three independent experiments; three to six technical replicates). Significance was assessed by a Kruskal–Wallis test followed by Dunn’s multiple comparisons test (**P < 0.01, ****P < 0.0001). (*B*) Immunofluorescence images after lentivirus treatment: PGC1α in yellow, alpha actinin (ACTN2) in red, and DAPI in blue. Scale bar: 20 µm. (*C*) mRNA expression of OXPHOS-related genes after lentivirus treatment. The dots represent technical replicates, while the color of each dot indicates the different batches of differentiation (three independent experiments; three to six technical replicates). Significance was assessed by an ordinary one-way ANOVA or Kruskal–Wallis test when data were not normally distributed followed by Šidák’s or Dunn’s multiple comparisons test respectively (*P < 0.05, **P < 0.01, ***P < 0.001, ****P < 0.0001, ns, not significant). (*D*) Schematic representation showing the experimental strategy for EHM casting after lentivirus treatment. Created in Biorender (https://BioRender.com/95dtsmq). (*E*) Trendline showing percentage of shortening for *PKP2^c.2013delC/WT^* and *PKP2^WT/WT^* EHM after lentivirus transduction at different time points. (*F*) Graph summarizing shortening for *PKP2^c.2013delC/WT^* and *PKP2^WT/WT^* EHM on day 42 (2–13 technical replicates). Significance was assessed by a Kruskal–Wallis test followed by Dunn’s multiple comparisons test (*P < 0.05).

As *PPARGC1A* has previously been shown to influence metabolic pathways and genes related to glycolysis and fatty acid metabolism were downregulated after knockdown of *PKP2*, we investigated whether *PPARGC1A* overexpression could modulate these pathways. *CPT1A*, a key regulator of fatty acid oxidation, was upregulated in both *PKP2^c.2013delC/WT^* and *PKP2^WT/WT^* hiPSC-CMs following PPARGC1A overexpression (see Supplementary material online, *Figure S7B*). In contrast, expression of glycolytic genes *PKM* and *FABP3* increased only in *PKP2^WT/WT^* cells, while *PFKP* expression remained unchanged in both genotypes (see Supplementary material online, *Figure S7B*). These results suggest that *PPARGC1A* overexpression may exert broader regulatory effects on cardiac metabolism.

To study whether *PPARGC1A* overexpression can be coupled to functional changes in cardiomyocytes, we used EHM. It has been previously shown that *PKP2^c.2013delC/WT^* EHM shows functional changes 42 days after casting.^26^ Therefore, we transduced both the *PKP2^c.2013delC/WT^* and *PKP2^WT/WT^* hiPSC-CMs with either the *GFP* or *PPARGC1A* lentivirus for a week and analyzed contractility 42 days after casting (*Figure 5D*). Similar to previous reports, we observed decreased shortening and contraction force from 35 days after casting in *PKP2^c.2013delC/WT^* EHM compared to *PKP2^WT/WT^* EHM treated with GFP virus (*Figure 6E* and Supplementary material online, *Figure S7C*). Treatment with *PPARGC1A* lentivirus in *PKP2^c.2013delC/WT^* EHM resulted in a progressive improvement in contractility from day 35, reaching statistical significance on day 42 post-casting (*Figure 6E* and *F* and Supplementary material online, *Figure S7C* and *D*). Together, these findings show that induction of *PPARGC1A* expression can partially increase the expression of genes involved in OXPHOS and leads to an increased contractile function in the *PKP2^c.2013delC/WT^* cardiomyocytes.

## 4. Discussion

Although mitochondrial dysfunction has been implicated in heart failure, its role in ACM pathogenesis and the underlying molecular mechanisms remain poorly understood. By utilizing human *in vitro* models and human explanted hearts in combination with RNA-sequencing data, we here show that expression of *PKP2* is directly linked to mitochondrial function and dysregulation of mitochondrial pathways, potentially contributing to the exercise-induced disease progression in ACM.

In this study, we use hiPSC-CMs harboring pathogenic *PKP2* variants—resulting in haploinsufficient PKP2 levels and reduced expression of other desmosomal proteins—and demonstrate that they exhibit diminished levels of OXPHOS components. This phenotype was observed in cardiomyocytes from two independent *PKP2* mutant hiPSC lines and further corroborated in explanted heart tissue from four ACM patients with *PKP2* variants. These data are in line with previous studies, showing that mutations or loss of OXPHOS components can cause cardiomyopathy in humans and lead to heart failure in mice.^41,42^ Cardiac-specific deletion of *Ndufs4*, a gene encoding a subunit of Complex I, reduced Complex I activity in mouse hearts without affecting ATP production, mitochondrial morphology or number under baseline conditions but led to heart failure after stress induction.^41^ Similarly, in our *PKP2* mutant hiPSC-CMs with decreased levels of OXPHOS components, we observed lower mitochondrial capacity. These findings indicate that OXPHOS downregulation may not immediately impair energy production at rest but it compromises mitochondrial efficiency under energy demanding conditions, such as exercise. This could explain why some ACM patients remain asymptomatic, especially in the early stages of the disease, while athletes diagnosed with ACM experience earlier symptom onset and disease progression.

As we observed decreased expression of OXPHOS-related genes across all complexes in *PKP2* mutant hiPSC-CMs, we hypothesized this effect to be regulated through a general transcription factor. Using RNA-sequencing data from *PKP2^c.2013delC/WT^* hiPSC-CMs and healthy human hearts, we consistently identified *PPARGC1A* expression to be positively correlated with *PKP2*, which was further validated through dose-dependent *PKP2* knockdown experiments. *PPARGC1A*, a key transcription factor that is highly expressed in tissues with high energy consumption, such as the heart, has been reported to be an important regulator of OXPHOS.^38,43^  *Ppargc1a* knockout mouse hearts showed reduced expression of OXPHOS-related genes, lower mitochondrial enzymatic activity and impaired contractile function while the mitochondrial number remained unchanged, which is in line with our results in *PKP2* mutant hiPSC-CMs and after *PPARGC1A* knockdown in control hiPSC-CMs.^36^

PGC1α is known to be activated by exercise in the heart and skeletal muscle and its loss has been shown to accelerate cardiac dysfunction and heart failure following stress induction, similar to the *Ndufs4* knockout mice.^21,38,44,45^ Consistent with this, treatment with the exercise mimetic AICAR increased *PPARGC1A* expression in control hiPSC-CMs, but not in *PKP2* mutant cells, likely due in part to reduced AMPKα levels. These findings suggest that *PKP2* mutant cardiomyocytes fail to induce *PPARGC1A* expression in response to energetic stress, limiting their ability to meet increased energy demands. This impaired adaptability may contribute to maladaptive responses such as ROS generation and cell death, key features of ACM pathogenesis.^18,46–53^ Based on these findings, restoring or enhancing PGC1α signaling could improve mitochondrial adaptability and represent a promising therapeutic strategy in ACM.

To explore this therapeutic avenue, we overexpressed PPARGC1A in *PKP2^c.2013delC/WT^* hiPSC-CMs. This did not only induce the expression of OXPHOS-related genes but also improved contractile function, supporting a functional role for reduced PGC1α in the contractile impairment observed in the *PKP2* mutant cells. Besides its role in OXPHOS, PGC1α has been shown to be involved in other metabolic pathways, such as glycolysis and fatty acid metabolism.^38^ As *PKP2* deficiency reduced expression of genes involved in these pathways, and we observed partial induction of glycolytic and fatty acid metabolism genes following *PPARGC1A* overexpression, this suggests broader metabolic benefits beyond OXPHOS restoration. However, the precise role of these metabolic changes and their involvement in ACM pathophysiology remains to be clarified in future studies.

Here, we used lentivirus in *PKP2* mutant hiPSC-CMs to increase PPARGC1A expression in PKP2 mutant cells. However, inducing *PPARGC1A* might become harmful in the long run, as long-term induction of *PPARGC1A* can lead to dilated cardiomyopathy in mice and is linked to poor prognosis in cancer.^38,54–57^ Moreover, the timing of PGC1α overexpression has been shown to significantly influence cardiac outcome.^55^ In 3-month-old WT mice, cardiac-specific overexpression of PGC1α enhanced mitochondrial function and improved cardiac performance. However, in 12-month-old WT mice, increased levels of PGC1α accelerated cardiac aging and significantly reduced lifespan due to increased mitochondrial damage and ROS exposure. Although we did not observe any contractile dysfunction in the *PKP2^WT/WT^* EHM tissues after overexpressing PGC1α, these findings underscore the need for careful regulation of PGC1α levels when considering therapeutic strategies targeting *PPARGC1A*.

While our findings consistently show reduced PPARGC1A expression in cardiomyocytes with decreased PKP2 levels, and identify diminished AMPKα as a potential contributing factor, the precise molecular mechanism linking PKP2 to PGC1α regulation remains to be fully elucidated. In addition to being regulated by multiple kinases, PGC1α expression is also influenced by calcium signaling.^38^ Studies using *Pkp2* knockout mouse models have demonstrated that PKP2 loss disrupts the expression of calcium-handling genes and alters intracellular calcium dynamics.^58,59^ Given that calcium plays a role in regulating both PGC1α and mitochondrial homeostasis, these disturbances may also contribute to impaired PGC1α expression. However, the exact mechanism how PKP2 modulates PGC1α expression remains to be determined and warrants further investigation.

While our study primarily focuses on *PKP2* and its link to mitochondrial function via *PPARGC1A*, the observed phenotype may not be exclusive to *PKP2* variants. Both our findings and previous research have shown that the loss of a single desmosomal component can destabilize other desmosomal and intercalated disk proteins, leading to widespread dysregulation.^7,9,26,27,60,61^ Additionally, a recent study demonstrated that *DSP* mutant hiPSC-CMs exhibit reduced respiratory capacity and increased ROS signaling, driven by EPAS1 induction.^62^ Although the mechanisms underlying decreased mitochondrial function may differ, accumulating evidence suggests that it could be a broader feature of ACM. Further research is essential to determine whether reduced mitochondrial function represents a common pathological mechanism across various ACM variants.

In summary, our study identifies impaired mitochondrial adaptability as a key consequence of *PKP2* haploinsufficiency in ACM, likely driven by disrupted AMPK–PGC1α signaling. This deficit compromises the cardiomyocyte’s ability to meet increased energy demands under stress. We show that inducing PGC1α expression not only increases OXPHOS gene expression but also improves contractile function in *PKP2* mutant cells. These findings provide a mechanistic rationale for therapeutic strategies aimed at enhancing PGC1α signaling to restore mitochondrial function and delay disease progression in ACM.

Translational perspective Our findings uncover a PKP2–AMPK–PGC1α signaling axis that links haploinsufficient PKP2 levels to impaired mitochondrial adaptability in ACM. This mechanistic insight provides a potential explanation for why exercise accelerates disease progression in ACM patients. Targeting this axis by enhancing PGC1α expression represents a promising therapeutic strategy to delay or prevent disease progression in individuals carrying pathogenic *PKP2* variants.

## Supplementary Material

cvag141_Supplementary_Data

## Data Availability

The data underlying this article are available in the article and in its online supplementary material. The generated single-cell RNA sequencing data are deposited at NCBI Gene Expression Omnibus repository under the accession number GSE298539.
