# Supplementary material for: PKP2 orchestrates OXPHOS expression in cardiomyocytes via a PGC1α-dependent mechanism
Source: Cardiovasc Res. 2026 Oct 10;122(14):2024–38. doi: 10.1093/cvr/cvag141 (PMC13653674; doi:10.1093/cvr/cvag141)
Supplement: Supplementary file 1 [file cvag141_supplementary_data.zip › Supplementary_files_resub2 (1).docx]

**Supplementary materials**

PKP2 orchestrates OXPHOS expression in cardiomyocytes via a PGC1α dependent mechanism

Han et al.

Content
Supplementary figures S1-7
Supplementary tables 1-4
Supplementary methods

**Supplementary figure 1**

**Figure S1. Molecular and functional phenotyping of *PKP2^c.1849C>T/WT^* hiPSC-CMs.**

(A) mRNA expression of *PKP2* and the other desmosomal components in *PKP2^c.1849C>T/WT^* hiPSC-CMs. Data plotted as mean. The dots represent technical replicates, while the color of each dot indicates the different batches of differentiation (3 independent experiments; 5-6 technical replicates). Significance was assessed by a two-tailed unpaired Student’s t test or two-tailed Mann-Whitney test when data were not normally distributed (*p<0.05, ****p<0.0001, ns, not significant). (B) Immunofluorescence images of *PKP2^c.1849C>T/WT^* and *PKP2^WT/WT^* hiPSC-CMs stained with PKP2, DSP and JUP in red, cardiac troponin T (TNNT2) in green and DAPI in blue. Scale bar: 20µm. (C) Representative western blot of the desmosomal proteins PKP2, DSP and JUP. (D) Quantification of the protein levels of PKP2, DSP and JUP normalized to VIN. Data plotted as mean. The dots represent technical replicates, while the color of each dot indicates the different batches of differentiation (3 independent experiments; 3-6 technical replicates). Significance was assessed by a two-tailed unpaired Student’s t test (***p<0.001, ****p<0.0001, ns, not significant). (E) Gene expression analysis of OXPHOS related genes (CI: Complex I, CIII: Complex III, CIV: Complex IV)). Data plotted as mean. The dots represent technical replicates, while the color of each dot indicates the different batches of differentiation (4 independent experiments; 6 technical replicates). Significance was assessed by a two-tailed unpaired Student’s t test or two-tailed Mann-Whitney test when data were not normally distributed (*p<0.05). (F) Representative western blot of OXPHOS proteins from different complexes (CI: Complex I, CII: Complex II, CIII: Complex III, CV: Complex V). (G) Quantification of OXPHOS protein levels normalized to TOM20. Data plotted as mean. The dots represent technical replicates, while the color of each dot indicates the different batches of differentiation (2-3 independent experiments; 2-6 technical replicates). Significance was assessed by a two-tailed unpaired Student’s t test or two-tailed Mann-Whitney test when data were not normally distributed (*p<0.05, ****p<0.0001). (H) Oxygen consumption rate (OCR) of *PKP2^c.1849C>T/WT^* and *PKP2^WT/WT^* hiPSC-CMs during the mitostress test. (I) Quantification of basal, ATP-linked, maximal respiration and spare capacity of the mitostress test. Data plotted as mean. The dots represent technical replicates, while the color of each dot indicates the different batches of differentiation (3 independent experiments; 3-10 technical replicates). Significance was assessed by a two-tailed unpaired Student’s t test or two-tailed Mann-Whitney test when data were not normally distributed (*p<0.05, ns, not significant).

**Supplementary figure 2**

**Figure S2. Expression of desmosomal proteins and OXPHOS related genes after knockdown of *PKP2* in control hiPSC-CMs.**

(A) Representative western blot of the desmosomal proteins PKP2, DSP and JUP after 5 days of si-*PKP2* treatment. (B) Quantification of the PKP2, DSP and JUP protein levels after 5 days of si-*PKP2* treatment normalized to VIN. Data plotted as mean. The dots represent technical replicates, while the color of each dot indicates the different batches of differentiation (3 independent experiments; 5-6 technical replicates). Significance was assessed by an ordinary one-way ANOVA followed by Dunnett’s multiple comparisons test (*p<0.05, ***p<0.001, ****p<0.0001, ns, not significant). (C) mRNA expression of OXPHOS related genes from different complexes after 5 days of si-*PKP2* treatment (CI: Complex I, CIII: Complex III, CIV: Complex IV, CV: Complex V). Data plotted as mean. The dots represent technical replicates, while the color of each dot indicates the different batches of differentiation (3 independent experiments; 3-6 technical replicates). Significance was assessed by an ordinary one-way ANOVA followed by Dunnett’s multiple comparisons test (*p<0.05, **p<0.01, ***p < 0.001, ****p<0.0001, ns, not significant).

**Supplementary figure 3**

**Figure S3. Expression of cytoskeletal proteins in *PKP2^c.2013delC/WT^* hiPSC-CMs.**
(A) Representative western blot of DES. (B) Quantification of DES protein relative to VIN. Data plotted as mean. The dots represent technical replicates, while the color of each dot indicates the different batches of differentiation (2 independent experiments; 6 technical replicates). Significance was assessed by a two-tailed unpaired Student’s t test (*p<0.05). (C) mRNA expression of *DES* after 5 days of si-*PKP2* treatment. Data plotted as mean. The dots represent technical replicates, while the color of each dot indicates the different batches of differentiation (3 independent experiments; 3-6 technical replicates). Kruskal-Wallis test followed by Dunn’s multiple comparisons test ****p<0.0001, ns, not significant). (D) mRNA expression of *DES* and OXPHOS related genes after knockdown of *DES* using siRNA. Data plotted as mean. The dots represent technical replicates, while the color of each dot indicates the different batches of differentiation (2 independent experiments; 6 technical replicates). Significance was assessed by a two-tailed unpaired Student’s t test or two-tailed Mann-Whitney test when data were not normally distributed (*p<0.05, ****p<0.0001, ns, not significant). (E) Immunofluorescence images of *PKP2^c.2013delC/WT^* and *PKP2^WT/WT^* hiPSC-CMs stained with α-tubulin (αTUB) and filamentous actin (Phalloidin) in yellow, cardiac troponin T (TNNT2) in green and DAPI in blue. Scale bar: 20µm.

**Supplementary figure 4**

**Figure S4. Further analysis of the single-cell sequencing data set of the *PKP2^c.2013delC/WT^* and *PKP2^WTWT^* hiPSC-CMs and expression of metabolic genes after knockdown of *PKP2* in control hiPSC-CMs.**

(A) Graph showing the relative contribution of *PKP2^c.2013delC/WT^* and *PKP2^WTWT^* hiPSC-CMs to each cluster. (B) Violin plot showing the expression of *DSP, JUP, DSG2* and *DSC2* in *PKP2^c.2013delC/WT^* hiPSC-CMs compared to control. (C) UMAP showing the expression pattern of desmosomal genes based on clustering of the cells. (D) KEGG enriched pathway analysis of the differentially expressed genes in *PKP2^c.2013delC/WT^* hiPSC-CMs compared to control of the single-cell RNA sequencing data set. (E) Graph showing more *PKP2* correlated genes related to metabolism and sarcomere. mRNA expression of genes related to glycolysis (F) and fatty acid metabolism (G) after 5 days of si-*PKP2* treatment. Significance was assessed by an ordinary one-way ANOVA or Kruskal-Wallis test when data were not normally distributed followed by Dunnett’s or Dunn’s multiple comparisons test respectively (*p<0.05, **p<0.01, ***p<0.001, ****p<0.0001, ns, not significant).

**Supplementary figure 5**

**Figure S5. Expression of OXPHOS related genes after knockdown of PPARGC1A.**

(A) mRNA expression of *PPARGC1A* 4 days after treatment of control hiPSC-CMs with 10nM siRNA against *PPARGC1A*. Data plotted as mean. The dots represent technical replicates, while the color of each dot indicates the different batches of differentiation (3 independent experiments; 5-6 technical replicates). Significance was assessed by a two-tailed Mann-Whitney test (****p<0.0001). (B) Immunofluorescence images of control hiPSC-CMs 4 days after treatment with si-*PPARGC1A*. Scale bar: 20µm. (C-F) Gene expression of OXPHOS related genes from complex I-V after si-*PPARGC1A* knockdown. Data plotted as mean. The dots represent technical replicates, while the color of each dot indicates the different batches of differentiation (3 independent experiments; 5-6 technical replicates). Significance was assessed by a two-tailed unpaired Student’s t test or two-tailed Mann-Whitney test when data were not normally distributed (*p<0.05, **p<0.01, ***p<0.001, ****p<0.0001, ns, not significant)

**Supplementary figure 6**

**Figure S6. Further analysis of the GTEx data set and localization of PGC1α in human explanted ACM hearts carrying different *PKP2* variants.**

(A) Graph showing the read counts of *PPARGC1A* across the healthy human heart samples taken from the GTEx data set. (B) Graph showing *PPARGC1A* correlated genes related to OXPHOS. (C) Graph showing *PKP2* correlated genes related to sarcomere and glycolysis*.* (D) Immunofluorescence images of left ventricular tissue collected from an explanted control and three ACM hearts stained with PGC1α in red, cardiac troponin T (TNNT2) in green and DAPI in blue. Scalebar: 100μm.

**Supplementary figure 7**

**Figure S7. Expression of other OXPHOS related genes, metabolic genes and contractility after PPARGC1A overexpression using lentivirus.**

(A) Gene expression of OXPHOS components from complex I-V after 1 week of lenti-PPARGC1A treatment in *PKP2^c.2013delC/WT^* and *PKP2^WT/WT^* hiPSC-CMs. Data plotted as mean. The dots represent technical replicates, while the color of each dot indicates the different batches of differentiation (2-3 independent experiments; 3-6 technical replicates). Significance was assessed by a Kruskal-Wallis test followed by Dunn’s multiple comparisons test (*p<0.05, **p<0.01, ns, not significant). (B) Gene expression of genes related to glycolysis and fatty acid metabolism after lentivirus treatment in *PKP2^c.2013delC/WT^* and *PKP2^WT/WT^* hiPSC-CMs. Data plotted as mean. The dots represent technical replicates, while the color of each dot indicates the different batches of differentiation (3 independent experiments; 3-6 technical replicates). Significance was assessed by an ordinary one-way ANOVA or Kruskal-Wallis test when data were not normally distributed followed by Šidák’s or Dunn’s multiple comparisons test respectively (*p<0.05, ****p<0.0001, ns, not significant). (C) Trendline showing absolute force of contraction for *PKP2^c.2013delC/WT^* and *PKP2^WT/WT^* EHM after lentivirus transduction at different time points. (D) Graph summarizing absolute force of contraction for *PKP2^c.2013delC/WT^* and *PKP2^WT/WT^* EHM on day 42 (2-13 technical replicates). Significance was assessed by a Kruskal-Wallis test followed by Dunn’s multiple comparisons test (*<p<0.05).

**Table S1. Differentially expressed genes related to the KEGG term oxidative phosphorylation and *PPARGC1A* expression**

| MeanExpression | SYMBOL | log2FoldChange | padj |
| --- | --- | --- | --- |
| 7374.19378 | UQCRC2 | -0.1079385 | 0.00393527 |
| 4854.30291 | NDUFS2 | -0.1111916 | 0.00825277 |
| 1761.45505 | ATP6V1B2 | -0.1131029 | 0.03866042 |
| 2625.6249 | ATP6V1E1 | -0.1201089 | 0.0316923 |
| 4969.02607 | SDHA | -0.1205953 | 0.00890101 |
| 5218.95141 | NDUFS5 | -0.1220941 | 0.01456384 |
| 905.03556 | SDHC | -0.132116 | 0.0448878 |
| 1018.93863 | PPA2 | -0.1376264 | 0.04033261 |
| 2586.95866 | ATP5ME | -0.1426313 | 0.01826928 |
| 2284.53623 | ATP6V0B | -0.1442116 | 0.00931795 |
| 7456.50152 | COX7C | -0.1448352 | 0.00057322 |
| 3765.23936 | NDUFV1 | -0.1476823 | 0.00574358 |
| 6027.53498 | ATP5F1C | -0.1514902 | 0.00036862 |
| 4897.37632 | ATP5PB | -0.1516573 | 0.00017221 |
| 2891.36929 | NDUFA1 | -0.1520048 | 0.00469131 |
| 1739.80902 | ATP6V1F | -0.1540937 | 0.00890741 |
| 5455.7738 | ATP5PF | -0.1561726 | 0.00052081 |
| 5009.07823 | COX5A | -0.1565716 | 0.00107018 |
| 4135.818 | UQCRB | -0.160655 | 0.00215157 |
| 7740.71062 | NDUFA4 | -0.1652451 | 8.77E-06 |
| 1469.04319 | ATP6V1C1 | -0.16594 | 0.00297882 |
| 1256.58135 | NDUFC1 | -0.1683336 | 0.00478257 |
| 2390.31586 | ATP6V1D | -0.169094 | 0.00035156 |
| 3215.21045 | ATP6AP1 | -0.1753515 | 0.0002206 |
| 1594.54413 | NDUFB1 | -0.1871653 | 0.00098451 |
| 4089.89965 | ATP5PD | -0.189245 | 0.00039765 |
| 3063.92746 | NDUFA6 | -0.1906847 | 6.90E-05 |
| 31393.5209 | ATP5F1B | -0.1955896 | 7.50E-09 |
| 3098.82528 | COX7A2 | -0.2019312 | 1.87E-06 |
| 4678.45926 | ATP5MG | -0.2037804 | 2.10E-06 |
| 593.139304 | ATP6V0A2 | -0.2048184 | 0.00894933 |
| 3144.57581 | UQCRFS1 | -0.2080402 | 1.07E-05 |
| 3484.31127 | COX8A | -0.2128138 | 0.00028909 |
| 5797.29667 | COX7B | -0.2133809 | 1.42E-06 |
| 6937.00684 | UQCRC1 | -0.2159706 | 8.56E-06 |
| 2006.60177 | SDHB | -0.21622 | 2.34E-05 |
| 2879.26648 | CYC1 | -0.2180522 | 0.00038938 |
| 1970.7509 | NDUFS8 | -0.218209 | 0.00085907 |
| 4950.7536 | NDUFS1 | -0.2214689 | 8.41E-08 |
| 4846.47761 | ATP5F1E | -0.2241952 | 2.40E-05 |
| 4561.86071 | UQCRH | -0.2263802 | 3.40E-06 |
| 5497.92847 | COX6B1 | -0.2276488 | 2.56E-05 |
| 1530.28616 | NDUFA3 | -0.2375945 | 0.00105597 |
| 2239.39449 | NDUFA8 | -0.2381368 | 2.03E-06 |
| 11068.577 | ATP5MC3 | -0.2460008 | 3.05E-11 |
| 17602.3232 | ND3 | -0.2499973 | 0.03572748 |
| 1353.9797 | COX6A1 | -0.2576014 | 1.76E-05 |
| 1407.16159 | NDUFB6 | -0.2644091 | 1.21E-06 |
| 3492.42813 | UQCR10 | -0.2660539 | 2.60E-06 |
| 178.928565 | NDUFA13 | -0.2683912 | 0.03580101 |
| 695.016586 | COX17 | -0.2994182 | 5.97E-05 |
| 2057.97897 | NDUFS6 | -0.3033267 | 2.68E-07 |
| 2861.8353 | NDUFB9 | -0.3041652 | 1.85E-09 |
| 1739.08081 | ATP5MF | -0.3107521 | 1.76E-08 |
| 472.338028 | NDUFB8 | -0.3120045 | 9.95E-05 |
| 1342.69337 | NDUFV3 | -0.3142837 | 2.50E-08 |
| 1073.81296 | ATP6V0A1 | -0.3236212 | 2.31E-09 |
| 433.7049 | NDUFA9 | -0.3298645 | 4.85E-05 |
| 2619.2847 | NDUFA12 | -0.3347741 | 1.80E-11 |
| 1432.43789 | NDUFB3 | -0.3440773 | 2.75E-09 |
| 2334.13016 | ATP5MC1 | -0.3712599 | 2.92E-15 |
| 342.593312 | UQCRHL | -0.3883755 | 1.85E-05 |
| 2364.28929 | NDUFB2 | -0.4257763 | 5.72E-20 |
| 4354.54297 | UQCRQ | -0.5366228 | 1.18E-30 |
| 532.264554 | ND6 | -0.5551158 | 0.00605444 |
| 1978.49192 | PPARGC1A | -0.2585024 | 5.44E-07 |

**Table S2. Clinical data of the explanted hearts from ACM patients.**

| **Gene Mutation** | **Protein Change** | **Sex** | **Age at Transplant** | **Initial Clinical Diagnosis** | **End-Stage Heart Failure** | **ECG** | **Sustained ventricular arrhythmias** | **MCS before transplant** |
| --- | --- | --- | --- | --- | --- | --- | --- | --- |
| *PKP2 c.235C>T* | p.Arg79X | M | 61 | ACM | Yes | - SR - Left axis - Interventricular conduction delay | VT | None |
| *PKP2 c.2386T>C* | p.Cys796Arg | F | 56 | ACM | Yes | - SR - Microvoltages - Negative T-waves V1-6 | VT | None |
| *PKP2 c.397C>T* | p.Gln133X | M | 66 | ACM | Yes | - SR - Microvoltages - Negative T-waves V1-4 - Ventricular extrasystoles | VF | None |
| *PKP2 c.2544G>A* | p.Trp848X | M | 47 | ACM | Yes | - Atrial pacing - Interventricular conduction delay - Microvoltages - Negative T-waves V1-5 | VT | None |

**Table S3. Quantitative PCR primers**

| Gene | Forward sequence (5’->3’) | Reverse sequence (5’->3’) |
| --- | --- | --- |
| *PKP2* | TGCTAAAGGCTGGCACAA | TAATCGCTGTGCGTGTAGTG |
| *JUP* | ACCAGGAGAGCAAGCTGAT | CTCCACAATGGCAGGCTTATT |
| *DSP* | GCACCAGCAGGATGTACTATT | TCAATTCAGGCTGCACGAT |
| *NDUFA4* | AAGCATCCGAGCTTGATCCC | ACAATGCCAGACGCAAGAGA |
| *NDUFB2* | GACCAGATCCCAGGTGTTCC | ATACGGAAAGTGACCCAGCAC |
| *NDUFB8* | TAGGACCCCAGAAGAACGGG | GTAGTCGCCATACCCCATGC |
| *NDUFA13* | GCGCCTACAAATCGAGGACT | ATCTGCAAGGTCCTCCGGTC |
| *NDUFS6* | GTAGGTCGTCAGAAAGAGGTGA | GCACGCTATCACCCGAGTC |
| *NDUFS8* | GCCACCATCAACTACCCGT | CTCGATGGTGATGGCCTGGG |
| *UQCRQ* | TCAGCTACAGCTTGTCACCG | GCGCAGAACATTGGGGATTC |
| *UQCRH* | CGAGGGACCATTGCGTGG | CCCAGATGATGAAGACTGGGG |
| *UQCRFS1* | GGAGCAGCCTGTGTTGGAC | AACAGAAGCAGGGACATTGAGG |
| *COX5A* | CATTGATGCTGCTTTGCGGG | AGGTCCTGCTTTGTCCTTAACA |
| *COX6A1* | TTGGTGTGTCCTCGGTTTCTC | AGTCTTCCACATGCGAGCTGA |
| *COX6B1* | CCGGGGTGCCTTTAGGATT | AAGGGGCGGTCTTGTAGTTC |
| *COX17* | GCGCGATGCGTGTATCATC | ATTCACACAGCAGACCACCA |
| *AT5F1B* | TTGCGGAGACTCACCCCTTC | CCGCATAGTCCCTGACAGGATG |
| *ATP5PF* | AGTCCATTTGCGGAGGAACA | TCCTCCAGATGTCTGTCGCTT |
| *ATP5ME* | GAGCCACGCGCTACAATTAC | GTCGCAGGGTCACTCACTTT |
| *PPARGC1A* | CAATTGAAGAGCGCCGTGTG | TGAAACCATAGCTGTCTCCATCA |
| *DES* | CTCCCCATCCAGACCTACTCT | GGTATGGACCTCAGAACCCCT |
| *PKM* | AAGTTAGGAAGGTCCTGGGAGA | TAGATCACCACGAGCCACCAT |
| *PFKP* | GACGACACTGTGGGTACCTG | AGCCTTCCTCTGGTGGAGAT |
| *ALDOA* | GACACTCTACCAGAAGGCGG | CACGCCCTTGTCTACCTTGA |
| *CPT1A* | GATTTTGCTGTCGGTCTTGG | CTCTTGCTGCCTGAATGTGA |
| *CPT1B* | ATCTACCTTCGAGGCAGGAG | GTCCAGTTTACGGCGATACA |
| *CD36* | CTTTGGCTTAATGAGACTGGGAC | GCAACAAACATCACCACACC |
| *FABP3* | GTGGAGTTCGATGAGACAACAGC | TGGTCTCTTGCCCGTCCCATTT |
| *GUS* | CCACCTAGAATCTGCTGGCTAC | GTGCCCGTAGTCGTGATACCAA |
| *HARP* | CACCATTGAAATCCTGAGTGATGT | TGACCAGCCCAAAGGAGAAG |
| *RPL32* | CAACGTCAAGGAGCTGGAAG | TGGGGTTGGTGACTCTGATG |

**Table S4. Antibodies and dilutions**

| Target | Dilution/Application | Company | Identifier |
| --- | --- | --- | --- |
| Plakophilin-2 (PKP2) | 1:10 – ICC | Progen | 651167 |
| Plakophilin-2 (PKP2) | 1:1000 – IB | BD Biosciences | AB_398109 |
| Desmoplakin (DSP) | 1:1000 – IB | Abcam | AB_443375 |
| Desmoplakin (DSP) | 1:50 – ICC | Progen | 65146 |
| Plakoglobin (γ-catenin) | 1:500 – ICC  1:1000 – IB | Santa Cruz | sc-398183 |
| Vinculin | 1:1000 – IB | Santa Cruz | AB_628438 |
| PGC1α | 1:500 – ICC | Novus | AB_1522118 |
| Total OXPHOS Rodent WB Antibody Cocktail | 1:1000 – IB | Abcam | AB_2629281 |
| TOM20 | 1:1000 – IB | Cell Signaling | AB_2687663 |
| Cardiac troponin T | 1:2000 – ICC (hiPSC-CMs) | Abcam | AB_956386 |
| Cardiac troponin T | 1:200 – ICC (human tissue) | Abcam | AB_306445 |
| Alpha actinin | 1:800 – ICC | Sigma-Aldrich | AB_476766 |
| phospho-AMPKα (Thr172) | 1:1000 – IB | Cell Signaling | AB_331250 |
| AMPKα | 1:1000 – IB | Cell signaling | #2532 |
| Desmin | 1:1000 – IB | R&D systems | AB_2092419 |
| α-tubulin | 1:2000 – ICC | Sigma-Aldrich | AB_477579 |
| Phalloidin | 1:1000 – ICC | Thermo Fisher | A12379 |

IB: Immunoblotting, ICC: immunocytochemistry

**Supplementary methods**

**Experimental model and study participants**

This study is part of the UCC-UNRAVEL biobank, a single-center research data platform that combines routine electronic health records enriched with deep phenotyping, genetic data and standardized biobanking to facilitate research in (inherited) heart diseases (www.unravelrdp.nl).^1^ The study was approved by the local institutional ethics review board (University Medical Center Utrecht, protocol UCC-UNRAVEL #12-387) and conforms to the principles outlined in the Declaration of Helsinki. Informed consent was obtained or in certain cases waived by the ethics committee when obtaining informed consent was not possible due to death of the patient.

**hiPSC culture**

The hiPSCs were cultured on Geltrex™ LDEV-Free, hESC-Qualified, Reduced Growth Factor Basement Membrane Matrix-coated wells (Gibco: A1413302) in Essential 8™ Medium (Gibco: A1517001). For passaging, cells were dissociated with TrypLE Express Enzyme (Gibco: 12605010) and resuspended in Essential 8™ Medium supplemented with 2μM Thiazovivin.

**hiPSC-cardiomyocyte differentiation, expansion and culture**

For directed differentiation to cardiomyocytes, hiPSCs were cultured until they reached 80-90% cell confluency. Differentiation was started (day 0) using RPMI-1640-Medium-GlutaMAX™ Supplement-HEPES (Gibco: 72400021) supplemented with 4µM CHIR99021, 0.5 mg/mL human recombinant albumin and 0.2 mg/mL L-Ascorbic Acid 2-Phosphate. After 48 hours, the medium was aspirated and cells were incubated in RPMI-1640-Medium-GlutaMAX™ Supplement-HEPES (Gibco: 72400021) supplemented with 5µM IWP2, 0.5 mg/mL human recombinant albumin and 0.2 mg/mL L-Ascorbic Acid 2-Phosphate for another 48 hours. On day 4 and 6 the medium was changed to RPMI-1640-Medium-GlutaMAX™ Supplement-HEPES (Gibco: 72400021) supplemented with 0.5 mg/mL human recombinant albumin and 0.2 mg/mL L-Ascorbic Acid 2-Phosphate. On day 8, cells were refreshed with RPMI-1640-Medium-GlutaMAX™ Supplement-HEPES (Gibco: 72400021) supplemented with B-27™Supplement (50x)-serum free (Gibco: 17504001). On day 10, cardiomyocytes were selected using RPMI 1640 Medium, no glucose, no glutamine (Biological Industries: 01-101-1A) supplemented with B-27™Supplement (50x)-serum free (Gibco: 17504001) and 4µM Lactate/HEPES for 4 days. After selection the cardiomyocytes were refreshed with RPMI-1640-Medium-GlutaMAX™ Supplement-HEPES (Gibco: 72400021) supplemented with B-27™Supplement (50x)-serum free (Gibco: 17504001). On day 16 the cells were dissociated using TrypLE™ Select Enzyme (10x), no phenol red (Gibco: A1217703) and seeded in 1:10 ratio in RPMI-1640-Medium-GlutaMAX™ Supplement-HEPES (Gibco: 72400021) supplemented with B-27™Supplement (50x)-serum free (Gibco: 17504001), 2μM Thiazovivin and 10% KnockOut™ Serum Replacement (Gibco: 10828028). The day after the cells were incubated in RPMI-1640-Medium-GlutaMAX™ Supplement-HEPES (Gibco: 72400021) supplemented with B-27™Supplement (50x)-serum free (Gibco: 17504001) and 5µM CHIR99021 to expand them for 4 days and then the cardiomyocytes were kept in RPMI-1640-Medium-GlutaMAX™ Supplement-HEPES (Gibco: 72400021) supplemented with B-27™Supplement (50x)-serum free (Gibco: 17504001) until they were used for experiments.

**Quantitative qPCR**

Total RNA was isolated from hiPSC-CMs using TRIzol™ (ThermoFisher: 15596018) or RNeasy Mini Kit (Qiagen: 74104) according to the manufacturer’s protocol. Total RNA was reverse transcribed using the iScript™ cDNA Synthesis Kit (BioRad: 1708891) and quantitative PCR (qPCR) reactions were performed on a Bio Rad CFX96 Real-Time PCR Detection System using the iQ SYBR Green Supermix kit (BioRad: 1708885). The ΔΔCt-method was used to analyze the data and the sequences of the primers used for amplification can be found in Table S3.

**Immunoblotting**

Protein was extracted from hiPSC-CMs using RIPA lysis buffer (10 mM Tris-pH7.5, 150 mM NaCl, 0.1% SDS, 0.5% sodium deoxycholate, 1%Triton X-100) supplemented with one tablet of cOmplete™ EDTA-free Protease Inhibitor Cocktail (Roche: 11836170001) and one tablet of PhosSTOP (Roche: 4906837001) per 10 mL of RIPA buffer. SDS–polyacrylamide gel electrophoresis and Western blot were performed using 10-15 µg of the protein extract. Membranes were blocked in 5% nonfat dry milk and incubated with the primary antibody overnight at 4 degrees. The next day the membrane was incubated with the corresponding secondary antibody coupled to horseradish peroxidase (1:10 000) for 45 minutes before being imaged using the Clarity™ Western ECL Substrate kit (BioRad: 1705061) and an Amersham Imager 680RGB device (GE Healthcare: 29270772). Protein quantification was performed using ImageJ. Antibodies and the dilutions used can be found in Table S4.

**Knockdown experiments**

Three-week-old hiPSC-CMs were dissociated using TrypLE™ Select Enzyme (10x), no phenol red and seeded in Geltrex-coated wells with RPMI-1640-Medium-GlutaMAX™Supplement-HEPES supplemented with B-27™Supplement (50x)-serum free (GIBCO: 17504001), 2μM Thiazovivin and 10% KnockOut™ Serum Replacement (Gibco: 10828028). After 24h, medium was refreshed with RPMI-1640-Medium-GlutaMAX™Supplement-HEPES supplemented with B-27™ Supplement (50x)-serum free. Five to seven days after reseeding, cells were transfected with either 10 nM scramble or siRNA oligo duplexes targeting *PKP2* (OriGene: SR303544), *PPARGC1A* (OriGene: SR323265) or *DES* (OriGene: SR301187) utilizing Lipofectamine™ 3000 Transfection Reagent (Thermo Fisher Scientific: L3000015) or Lipofectamine™ RNAiMAX (Thermo Fisher Scientific: 13778075) according to the manufacturers’ instructions.

**Seahorse assay**

hiPSC-CMs were plated at 60.000 cells per well in a 24-well cell culture microplate (Agilent, 100777-004). 30 minutes before running the XF Cell Mito Stress Test, the cells were incubated with Seahorse XF base medium (Agilent, 102353-100) supplemented with 10 mM glucose, 1 mM pyruvate, 2 mM glutamine. A XFe24 Analyzer (Agilent: 420017) was used to run the experiment with the following drug concentrations: 1 μM Oligomycin, 1 μM FCCP, Rotenone and Antimycin A 2.5 μM. For normalization, the CyQUANT™ Cell Proliferation Assay (Thermo Fisher Scientific: C7026) was used according to the manufacture’s protocol and the data was analyzed using the Seahorse Wave Desktop Software.

**AICAR treatments**

Three-week-old hiPSC-CMs were dissociated using TrypLE™ Select Enzyme (10x), no phenol red and seeded in Geltrex-coated wells with RPMI-1640-Medium-GlutaMAX™Supplement-HEPES supplemented with B-27™Supplement (50x)-serum free (GIBCO: 17504001), 2μM Thiazovivin and 10% KnockOut™ Serum Replacement (Gibco: 10828028). After 24h, medium was refreshed with RPMI-1640-Medium-GlutaMAX™Supplement-HEPES supplemented with B-27™ Supplement (50x)-serum free. Five to seven days after reseeding, cells were treated with 500µM AICAR (Merck: A9978) for 15min to isolate protein and for 24 hours to isolate RNA. Protein and RNA were isolated as described above.

**Overexpression experiments**

hiPSC-CMs were dissociated using TrypLE™ Select Enzyme (10x), no phenol red and seeded in Geltrex-coated wells with RPMI-1640-Medium-GlutaMAX™Supplement-HEPES supplemented with B-27™Supplement (50x)-serum free (GIBCO: 17504001), 2μM Thiazovivin and 10% KnockOut™ Serum Replacement (Gibco: 10828028). After 24h, medium was refreshed with RPMI-1640-Medium-GlutaMAX™Supplement-HEPES supplemented with B-27™ Supplement (50x)-serum free. Five to seven days after reseeding, cells were infected with either PPAGRC1A or EGFP cloned into the pLVX-IRES-Hygro vector backbone (Clontech: 632185). After seven days, RNA and protein samples were collected (as described above). The viral particles used were harvested 48h after transfection of 7x10^6^ HEK293X cells with 11.9μg pLVX-PPARGC1A gene or 10µg pLVX-EGFP together with 9μg psPAX2 and 3μg pMD2G.

**Immunocytochemistry**

For the human explanted hearts, the samples were boiled for 20 minutes in sodium citrate (10 mM sodium citrate at pH 6.0), cooled down for about 30 minutes and washed with PBS. After incubating the sections in blocking buffer (0,5% TX100 + 0,5% BSA in PBS) for 1h, they were incubated with the primary antibody diluted in blocking buffer overnight at 4 ºC. After washing them three times with PBS-T (0.2% Tween20 in PBS) the corresponding secondary antibody diluted in blocking buffer (1:200) was added for 1h with DAPI. After three washes with PBS-T and one wash with water the sections were mounted with Mowiol (24% (w/v) Glycerol (Baker), 9.6% (w/v) Mowiol 4-88 (Calbiochem), 0.1 M Tris-HCl pH 8.5) and imaged using Olympus VS200 Research Slide Scanner.

For the hiPSC-CMs, the cells were cultured on Geltrex-coated coverslips and fixed in 4% paraformaldehyde for 30 min at RT. After three washing steps with PBS, cells were permeabilized with 0,1% Triton X-100 in PBS for 8 min at RT. Permeabilized cells were blocked with 4% goat-serum in PBS for an hour at RT and incubated with primary antibody diluted in 4% goat-serum overnight at 4°C. Subsequently, the cover slips were washed three times with PBS-T (0.05% Tween20 in PBS) and incubated with secondary antibody (1:200) and DAPI (1:1000) for an hour at RT. Then, cover slips were washed three times with PBS-T (0.05% Tween20 in PBS), one time with water and mounted with Mowiol on slides. The slides were then imaged with the Leica TCS SPE confocal microscope.

Antibodies and the dilutions used can be found in Table S4.

**EHM generation**

For generating EHM, the protocol published by Tiburcy et al was followed.^2^ In short, hiPSC-CMs were mixed together with human foreskin fibroblasts at a ratio of 70:30. The cell mixture was resuspended in Collagen type I (Collagen Solutions: FS22024) diluted into RPMI 2× (Thermo Fisher Scientific: 51800-035). The cell–collagen mixture was then added in each well of a 48 EHM multi-well plate (myriamed GmbH: myrPlate-TM5) and after 45 min at 37 °C EHM medium freshly supplemented with TGFβ1 was added. During the first three days after the casting process, the tissues were refreshed daily with EHM medium supplemented with TGFβ1. Then, the tissue medium was replaced daily with EHM medium until 2 weeks and after that with MEM alpha medium until the tissues were collected for molecular analyses.

**Contraction analyses**

Contraction measurements were performed using video-optic recordings of EHM mediated pole bending in a myrPlate-TM5 culture format at 37 °C. The spontaneously contracting EHM was recorded for at least 2 min at 50 fps at the indicated time points in a myrImager prototype (myriamed GmbH). Percent pole bending is reported as a surrogate for force of contraction.

**Bulk RNA sequencing**

The bulk RNA-sequencing dataset of *PKP2^c.2013delC/WT^* and its isogenic control *PKP2^WT/WT^* hiPSC-CMs in the GEO-database under accession GSE160289 has been used to analyze differential expression and KEGG pathway analysis. Differential expression was calculated using DESeq2 v1.2 with pooled dispersion estimates. Enrichment analysis of the differentially expressed genes was done using the STRINGv11.5 database. No changes were made to the default settings. Homo sapiens served as background. KEGG data sources were considered for enrichment analysis.

**Single-cell RNA sequencing**

hiPSC-CMs were dissociated as described above and single-cell sorted using the FACSJazz™ Cell Sorter (BD Biosciences) into 384-well plates provided by Single Cell Discoveries. After sorting, plates were centrifuged at 4 °C, put on dry ice and stored at -80 °C. Then the plates were prepared for single-cell mRNA sequencing by Single Cell Discoveries according to an adapted version of the SORT-seq protocol.^3^ Single-cell RNA sequencing data have been deposited at Gene Expression Omnibus under the accession number GSE298539.

**Single-cell data analysis**

Single-cell transcriptomics analysis was done using the Seurat package (version 5.1.0) mostly following the Seurat tutorial (https://satijalab.org/seurat/articles/pbmc3k_tutorial). In total, two 384 well plates with *PKP2^c.2013delC/WT^* hiPSC-CMs and two 384 well plates with *PKP2^WT/WT^* hiPSC-CMs were sequenced and analyzed. For downstream analysis, we included cells that have more than 2000 genes and between 4000 and 80000 transcripts and ended up with 647 *PKP2^c.2013delC/WT^* and 681 *PKP2^WT/WT^* cells. The median number of transcripts of the cells that were included in the analysis was 23932 and the median number of genes expressed in the cells was 5466. The dimensionality reduction was done based on the top 10 PCs, followed by using FindNeighbors and FindClusters function to cluster cells (resolution = 0.4).

**GTEx data analysis**

Adult bulk RNA sequencing data specific for left ventricular heart tissue, including the de-identified metadata, were obtained from the GTEx portal (V8 release) at gtexportal.org on 31/07/24. Exp ression data was downloaded as Transcripts per Million (TPM) and filtered for the target gene-of-interest (*PKP2* or *PPARGC1A*) for further downstream applications. A basic R script (build 4.2) was written to perform rank and correlation analyses for said genes.

**Quantification and statistical analysis**

Data are presented as the mean ± standard error of the mean (SEM) and analyzed using GraphPad Prism 10. First, outliers were identified using the ROUT method (Q=1%) and removed if present. Then the datapoints were checked for normality using the D'Agostino & Pearson test and Anderson-Darling test. If normally distributed, the results were assessed for significance using a two-tailed unpaired Student’s t test when comparing two groups or an ordinary one-way ANOVA when comparing more than two groups. If the data is not normally distributed, two-tailed Mann-Whitney test was used to assess significance when comparing two groups and Kruskal-Wallis test when comparing more than two groups.

**References:**

1.	Sammani A, Jansen M, Linschoten M, Bagheri A, Jonge N de, Kirkels H, Laake LW van, Vink A, Tintelen JP van, Dooijes D, Riele ASJM te, Harakalova M, Baas AF, Asselbergs FW. UNRAVEL: big data analytics research data platform to improve care of patients with cardiomyopathies using routine electronic health records and standardised biobanking. *Netherlands Heart Journal* 2019;27:426–434.

2.	Tiburcy M, Hudson JE, Balfanz P, Schlick S, Meyer T, Liao MLC, Levent E, Raad F, Zeidler S, Wingender E, Riegler J, Wang M, Gold JD, Kehat I, Wettwer E, Ravens U, Dierickx P, Laake LW Van, Goumans MJ, Khadjeh S, Toischer K, Hasenfuss G, Couture LA, Unger A, Linke WA, Araki T, Neel B, Keller G, Gepstein L, Wu JC, Zimmermann WH. Defined engineered human myocardium with advanced maturation for applications in heart failure modeling and repair. *Circulation* 2017;135:1832–1847.

3.	Muraro MJ, Dharmadhikari G, Grün D, Groen N, Dielen T, Jansen E, Gurp L van, Engelse MA, Carlotti F, Koning EJP de, Oudenaarden A van. A Single-Cell Transcriptome Atlas of the Human Pancreas. *Cell Syst* 2016;3:385-394.e3.
