# Supplementary material for: PKP2 orchestrates OXPHOS expression in cardiomyocytes via a PGC1α-dependent mechanism
Source: Cardiovasc Res. 2026 Oct 10;122(14):2024–38. doi: 10.1093/cvr/cvag141 (PMC13653674; doi:10.1093/cvr/cvag141)
Supplement: Supplementary file 1 [file cvag141_supplementary_data.zip › unedited_gels.docx]

Figure 1

Figure 2

Figure 4

Figure 5

Supplemental figure1

Supplemental figure 2

Supplemental figure 3
